# Blockage of Osteopontin‐Integrin *β*3 Signaling in Infrapatellar Fat Pad Attenuates Osteoarthritis in Mice

**DOI:** 10.1002/advs.202300897

**Published:** 2023-05-23

**Authors:** Bingyang Dai, Yuwei Zhu, Xu Li, Zuru Liang, Shunxiang Xu, Shian Zhang, Zhe Zhang, Shanshan Bai, Wenxue Tong, Mingde Cao, Ye Li, Xiaobo Zhu, Wei Liu, Yuantao Zhang, Liang Chang, Patrick Shu‐hang Yung, Kevin Ki‐wai Ho, Jiankun Xu, To Ngai, Ling Qin

**Affiliations:** ^1^ Musculoskeletal Research Laboratory of Department of Orthopaedics & Traumatology and Innovative Orthopaedic Biomaterial & Drug Translational Research Laboratory Li Ka Shing Institute of Health Sciences The Chinese University of Hong Kong Hong Kong 999077 China; ^2^ Areas of Excellence Centre for Musculoskeletal Degeneration and Regeneration Department of Orthopaedics and Traumatology Faculty of Medicine The Chinese University of Hong Kong China; ^3^ Department of Chemistry The Chinese University of Hong Kong Hong Kong 999077 China; ^4^ Department of Rehabilitation Sciences The Hong Kong Polytechnic University Hong Kong 999077 China

**Keywords:** infrapatellar fat pad, integrin *β*3, nanogel, osteoarthritis, osteopontin, siRNA

## Abstract

The knowledge of osteoarthritis (OA) has nowadays been extended from a focalized cartilage disorder to a multifactorial disease. Although recent investigations have reported that infrapatellar fat pad (IPFP) can trigger inflammation in the knee joint, the mechanisms behind the role of IPFP on knee OA progression remain to be defined. Here, dysregulated osteopontin (OPN) and integrin *β*3 signaling are found in the OA specimens of both human and mice. It is further demonstrated that IPFP‐derived OPN participates in OA progression, including activated matrix metallopeptidase 9 in chondrocyte hypertrophy and integrin *β*3 in IPFP fibrosis. Motivated by these findings, an injectable nanogel is fabricated to provide sustained release of siRNA *Cd61* (^RGD−^Nanogel/siRNA *Cd61*) that targets integrins. The ^RGD−^Nanogel possesses excellent biocompatibility and desired targeting abilities both in vitro and in vivo. Local injection of ^RGD−^Nanogel/siRNA *Cd61* robustly alleviates the cartilage degeneration, suppresses the advancement of tidemark, and reduces the subchondral trabecular bone mass in OA mice. Taken together, this study provides an avenue for developing ^RGD−^Nanogel/siRNA *Cd61* therapy to mitigate OA progression via blocking OPN‐integrin *β*3 signaling in IPFP.

## Graphical abstract

IPFP aberration‐associated OPN plays a crucial role in OA progression, including articular chondrocyte hypertrophy and IPFP fibrosis. The ^RGD‐^Nanogel/siRNA *Cd61* system effectively attenuates the cartilage erosion and IPFP inflammation, suppresses the advancement of tidemark, and reduces the subchondral trabecular bone mass, therefore may serve as an innovative therapeutic strategy to mitigate OA progression.

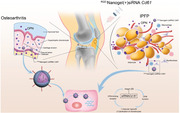

## 1 Introduction

Osteoarthritis (OA) is the most prevalent joint disease characterized by destruction of articular cartilage, pathological remodeling of subchondral bone, synovial inflammation, and osteophytes formation.^[^ ^1^, ^2^, ^3^, ^4^ ^]^ Current treatments include moderate exercise programs, topical NSAIDs, oral paracetamol, intra­articular injection of corticosteroids or hyaluronic acid, yet with limited effectiveness on preventing OA progression at late stage of OA.^[^ ^5^, ^6^, ^7^, ^8^ ^]^ Recent clinical studies have found that the alteration of magnetic resonance imaging (MRI) signal intensity of infrapatellar fat pad (IPFP) is associated with knee structural abnormalities and symptoms.^[^ ^9^, ^10^, ^11^ ^]^ Besides, the IPFP's volumetric and morphometric characteristics may also be important features during the progression of OA.^[^ ^11^ ^]^ Such pathological changes of IPFP in OA patients result in pain, fibrosis, and aberrant production of cytokines.^[^ ^11^, ^12^, ^13^, ^14^ ^]^ Fibrosis of IPFP alters the biomechanical properties, in terms of the capability of gravitational force absorption on the knee joint, accelerating OA progression.^[^ ^12^, ^14^ ^]^ Moreover, IPFP‐derived soluble cytokines, including interleukin‐6 (IL‐6), tumor necrosis factor (TNF), and visfatin, may induce destruction of the adjacent cartilage, synovial membrane, and subchondral bone.^[^ ^10^, ^15^, ^16^ ^]^ Although it has been proposed that the stimuli from IPFP may be involved in OA progression, further illustration for this aspect remains elusive.

Osteopontin (OPN) is an extracellular matrix protein, containing Arg‐Gly‐Asp (RGD), that can be secreted from synovial cells and stromal vascular fraction of adipose tissue where it favors cell adhesion, cell migration, and extracellular matrix fibrosis.^[^ ^17^, ^18^, ^19^ ^]^ OPN is crucial for the development of chronic inflammation by assembling macrophages in adipose tissue.^[^ ^17^, ^19^ ^]^ OPN is also essential for the differentiation and maturation of myofibroblasts and macrophages infiltration in response to the profibrotic circumstance, where myofibroblasts and macrophages express alpha‐smooth muscle actin (*α*SMA, a marker of myofibroblast), fibronectin, and collagen IV.^[^ ^20^, ^21^ ^]^ Although OPN belongs to a family of secreted acidic proteins which can modulate bone mineralization,^[^ ^19^, ^22^ ^]^ it also impairs articular cartilage in the rheumatoid arthritis model along with the enhanced angiogenesis and induction of apoptosis in chondrocyte,^[^ ^23^ ^]^ as well as with the increased matrix metallopeptidase 9 (MMP9) expression in OA patients.^[^ ^24^ ^]^ Specific inhibition of integrin *β*3, a core component of OPN receptor complexes, shows less cartilage degeneration and synovitis.^[^ ^25^ ^]^ To date, however, the effects of IPFP aberration‐associated OPN on articular joint homeostasis have yet to be characterized.

Articular cartilage is avascular and aneural, and synovial fluid is continuously exchanged for the joint environment, resulting in rapid clearance of drugs by the lymphatic system, yet without effective therapy.^[^ ^1^ ^]^ Owing to the closed compartment, local administration is favored for small‐interfering RNA (siRNA)‐based therapies. Several clinical trials based on siRNA technology have been conducted, including treatments for cancer, ocular, infectious diseases, hemophilia A or B, and rheumatic conditions.^[^ ^26^, ^27^, ^28^, ^29^, ^30^ ^]^ Besides, siRNA treatment of acute intermittent porphyria (givosiran) and polyneuropathy of hereditary TTR‐mediated amyloidosis (patisiran) have been approved by US Food and Drug Administration.^[^ ^31^, ^32^ ^]^ However, local injection of naked siRNA is susceptible to nuclease degradation and inefficiency in vivo administration.^[^ ^33^ ^]^ Thus, a delivery system is essential to overcome the obstacles.

In this study, we focused on the role of IPFP‐derived OPN in OA progression and further anchored siRNA *Cd61* (integrin *β*3) in IPFP, the site of early pathological change, for attenuating the OA progression. We developed a core/shell nanogel complex (^RGD−^Nanogel/siRNA *Cd61*) to carry siRNA *Cd61* that binds to the sites with rich expression of OPN‐receptors using RGD sequence. Intra‐IPFP injection of ^RGD−^Nanogel/siRNA *Cd61* attenuated cartilage degeneration and IPFP inflammation.

## 2 Results

### 2.1 The Pathological Alterations of Cartilage and IPFP in OA Mice

**Figure 1:**
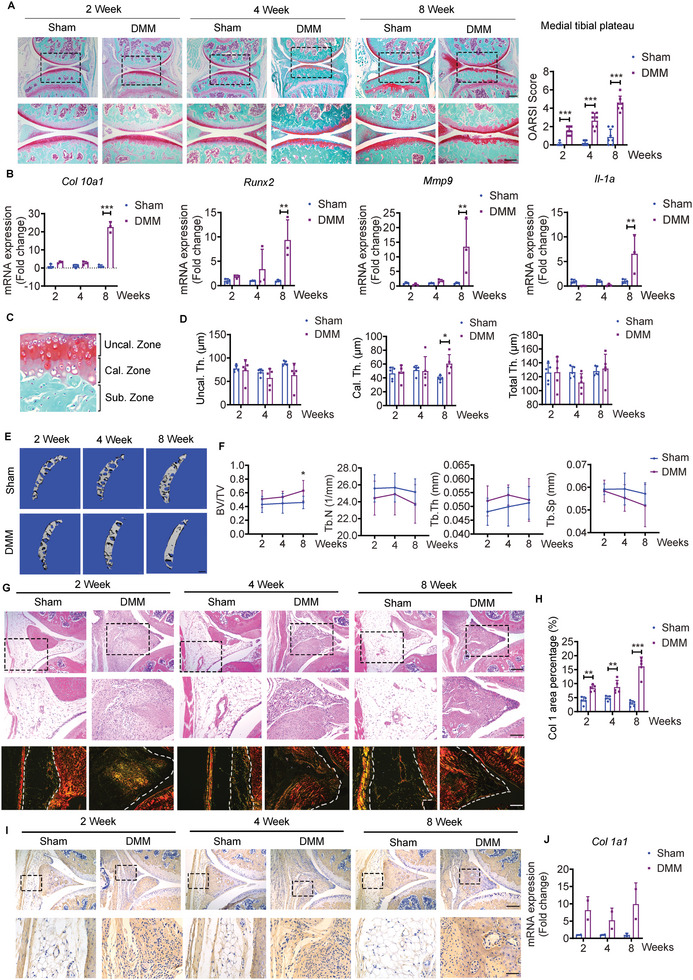
The pathological alterations of knee joint in osteoarthritis (OA) mice. A) Representative safranin O/fast green staining and quantifications of OA severity by OARSI scores of knee joint sections from sham and destabilization of the medial meniscus (DMM) mice at weeks 2, 4, and 8. Scale bar: 300 µm. The bottom row was magnified from the dotted frame in top row. *n* = 7 mice per group. Scale bar: 200 µm. B) Relative expression of *Col10a1*, *Runx2, Mmp9*, and *Il‐1a* in cartilage from sham and DMM mice at weeks 2, 4, and 8. *n* = 3 biologically independent samples per group, and each sample is assembled from four mice. C) Regions of interest (ROI) for uncalcified cartilage thickness (Uncal. Th.), calcified cartilage thickness (Cal. Th.), and total cartilage thickness (Total Th. = Uncal. Th. + Cal. Th.) in articular cartilage. D) Quantifications of average thicknesses of Uncal. Th., Cal. Th., and Total Th. from sham and DMM mice at weeks 2, 4, and 8. *n* = 5 mice per group. E) Representative µCT images and F) quantifications of subchondral trabecular bone from sham and DMM mice at weeks 2, 4, and 8. *n* = 7 mice per group. Scale bar: 200 µm. G,H) Representative H&E staining of IPFP (G) from sham and DMM mice at weeks 2, 4, and 8. Scale bar: 200 µm. The middle row (G) was magnified from the top row. Scale bar: 100 µm. Representative Picro‐Sirius red staining (G) and quantifications (H) of Col I area percentage of IPFP from sham and DMM mice at weeks 2, 4, and 8. *n* = 5 mice per group. Scale bar: 100 µm. I) Representative images of immunohistochemical staining of Col 1A1 in IPFP from sham and DMM mice at weeks 2, 4, and 8. Scale bar: 200 µm. The bottom row was magnified from the dotted frame in top row. Scale bar: 50 µm. J) Relative expression of *Col 1a1* in infrapatellar fat pad (IPFP) for sham and DMM mice. *n* = 2 biologically independent samples per group and each sample is assembled from six mice. Images are representative of 3 independent experiments. BV/TV: Bone volume fraction; Tb.Th: trabecular bone thickness; Tb.Sp: trabecular bone separation; and Tb.N: trabecular bone number. All data are presented as mean ± SD. Two‐way ANOVA with *Sidak's post hoc* test (A, B, D, F, H, and J) were used. *p < 0.05, **p < 0.01, and ***p < 0.001.

Mouse anterior medial meniscotibial ligament was transected to establish a destabilization of the medial meniscus (DMM) model. We observed a rough surface with small fibrillations in the articular cartilage of the medial tibial plateau at week 2, decreased proteoglycan staining (red) and small cartilage lesions across articular cartilage at week 4, and vertical clefts/erosion to the calcified cartilage extending over 50% of the articular surface at week 8 in DMM group (**Figure** **1**A). The histopathological grade and stage of DMM group were significantly higher than that of sham group at weeks 2, 4, and 8 post‐surgeries based on Osteoarthritis Research Society International (OARSI) assessment criteria (Figure 1A). The mRNA expression levels of chondrocyte hypertrophic markers were significantly higher in the cartilage of DMM group as compared to the sham group at week 8, as well as *Mmp9*, a class of enzyme that belongs to the zinc‐metalloproteinases family involved in the generation of hypertrophic chondrocyte,^[^ ^34^ ^]^ and *interleukin 1 alpha* (*Il‐1a*) (Figure 1B). Safranin O/fast green staining displayed the thickened calcified cartilage area in the DMM group at week 8 (Figure 1C,D). Bone volume fraction (BV/TV) was significantly higher in the DMM group than that in the sham group at week 8 (Figure 1E,F), echoing that increased bone mass of subchondral trabecular bone partially attributed to the skewing of calcified cartilage. These results suggest that the pathological alterations in articular cartilage may occur as early as at week 2 and are progressively over time in DMM mice.

DMM mice developed severe synovitis as displayed by thickened synovium and notable inflammatory infiltration quantified by synovitis score at weeks 2, 4, and 8 (Figure S1, Supporting Information). Compared to the IPFP next to the anterior medial meniscus in the sham group, compelling densification was found to replace the original resident adipocytes in the DMM group (Figure 1G). Picro Sirius red staining displayed substantial yellow/orange birefringence, referring to the hallmark of collagen I, in the DMM group at all time points (Figure 1G,H). Furthermore, immunohistochemical staining and mRNA expression confirmed that collagen 1A1 was enriched in IPFP of the DMM group, but almost blunted in the sham group (Figure 1I,J). These results indicate that the pathological alterations in IPFP may also occur as early as at week 2 in DMM mice.

### 2.2 Osteopontin Accumulates in Calcified Cartilage and IPFP

**Figure 2:**
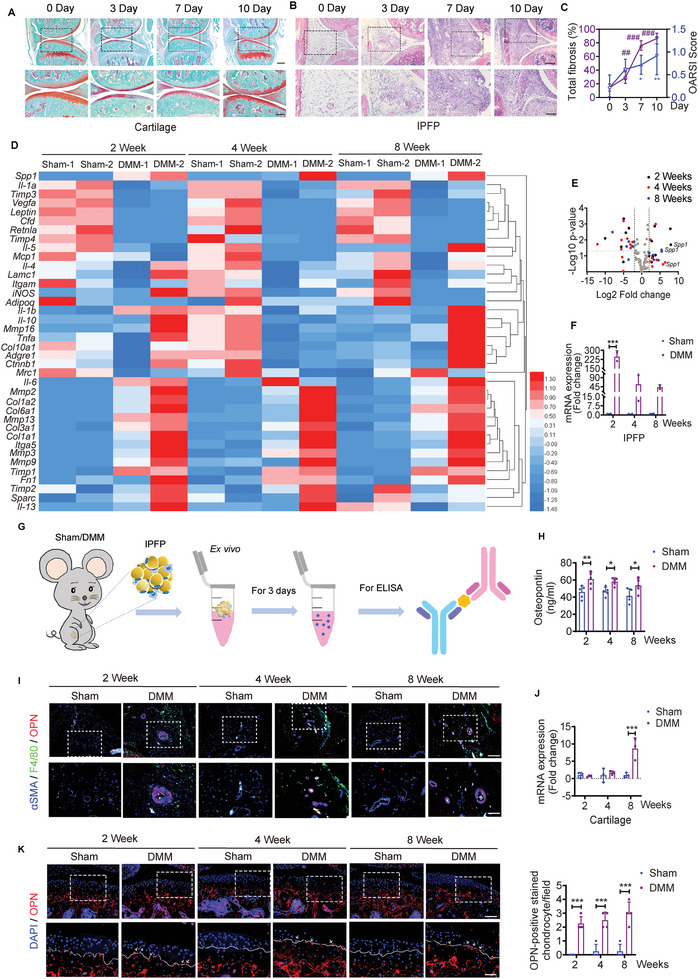
The expression levels of osteopontin (OPN) in cartilage and IPFP. A) Representative safranin O/fast green staining of knee joint sections from sham and DMM mice at days 0, 3, 7, and 10. Scale bar: 300 µm. The bottom row was magnified from the dotted frame in top row. Scale bar: 200 µm. B) Representative H&E staining of inflamed IPFP of knee joint sections from sham and DMM mice at days 0, 3, 7, and 10. Scale bar: 200 µm. The bottom row was magnified from the dotted frame in top row. Scale bar: 100 µm. C) Quantifications of OA severity by OARSI scores for articular cartilage and fibrosis scores for IPFP from sham and DMM mice at days 0, 3, 7, and 10. *n* = 5 mice per group. D) Heatmap summarizing the normalized fold changes in mRNA expression of different biomarkers in IPFP from sham and DMM mice at weeks 2, 4, and 8. *n* = 2 biologically independent samples per group, and each sample is assembled from six mice. E) Volcano plots of transcripts differentially expressed from IPFP (D) between sham and DMM mice. F) Relative expression of *Spp1* in IPFP from sham and DMM mice at weeks 2, 4, and 8. *n* = 2 biologically independent samples per group, and each sample is assembled from six mice. G) Schematic diagram illustrating the ELISA measuring concentrations of OPN in conditioned medium with isolated IPFP from sham or DMM mice. H) Free osteopontin (OPN) levels in the conditioned medium of isolated IPFP from sham or DMM mice at weeks 2, 4, and 8 post‐DMM or ‐sham surgery. *n* = 5 mice per group. I) Representative images of immunofluorescence staining of OPN, F4/80, and alpha‐smooth muscle actin (*α*SMA) in IPFP from sham and DMM mice at weeks 2, 4, and 8. Scale bar: 100 µm. The bottom row was magnified from the dotted frame in top row. Scale bar: 50 µm. J) Relative expression of *Spp1* in articular cartilage from sham and DMM mice at weeks 2, 4, and 8. *n* = 3 biologically independent samples per group, and each sample is assembled from four mice. K) Representative images and quantifications of immunofluorescence staining of OPN in articular cartilage from sham and DMM mice at weeks 2, 4, and 8. Scale bar: 100 µm. The bottom row was magnified from the dotted frame in the top row. *n* = 4 mice per group. Scale bar: 50 µm. Images are representative of 3 independent experiments. All data are presented as mean ± SD. One‐way ANOVA with *Dunnett's post hoc* test (C) and two‐way ANOVA with *Sidak's post hoc* test (F, H, J, and K) were used. *p < 0.05, **p < 0.01, and ***p < 0.001.

Given the concurrent pathological changes in cartilage surface and IPFP at week 2, we questioned if such alterations were triggers or resultant phenomenon of OA. To further assess the temporo‐spatial relationship of pathogenesis in cartilage and IPFP, we performed histological analysis at earlier time points after DMM surgery. Although the deteriorated articular cartilage integrity with a significant greater OARSI score arose at day 7 post‐surgery, IPFP displayed severe fibrosis at day 3 relative to the group at day 0 set as baseline (**Figure** **2**A–C). These data suggest that the fibrosis in IPFP predates the damage to the articular cartilage surface in DMM model.

The rapid formation of fibrosis in IPFP indicated the possibility that there might be involved an array of secretory cytokines from IPFP. We further characterized the mRNA expression in IPFP depots, including inflammation, extracellular matrix, matrix metallopeptidase, and lipogenesis. The *secreted phosphoprotein 1* (*spp1*, also known as *osteopontin*) was the most highly upregulated gene in IPFP of the DMM mice at all time points (Figure 2D). Although several other markers were statistically significant at one cohort, the magnitude fold changes of *spp1* from IPFP were differentially expressed at weeks 2 and 8 post‐surgery (Figure 2E). *spp1* was found to increase significantly in the DMM group, with roughly 200 folds at week 2, 11–100 folds at week 4, and 40 folds at week 8, as compared to that in the sham group (Figure 2F). Besides, the concentrations of OPN in conditioned medium measured by ELISA were significantly increased in IPFP of DMM mice than that of sham mice at weeks 2, 4, and 8 post‐DMM or ‐sham surgeries (Figure 2G,H).

We further characterized the correlations among OPN, macrophage, and myofibroblast in IPFP. Co‐staining of *α*SMA and F4/80, a marker of macrophage, showed that OPN mainly accumulated at the *α*SMA‐expressing regions in the DMM group, and F4/80 significantly enriched in the IPFP of each cohort (Figure 2I). However, the expression of *spp1* was similar between the sham and DMM groups in articular cartilage at weeks 2 and 4, and it was significantly higher in the DMM group as compared to the sham group at week 8 (Figure 2J). Immunofluorescence staining revealed that OPN expression was abundant in calcified cartilage and subchondral bone, while OPN‐positive stained chondrocytes overflowed the tidemark to the uncalcified cartilage in the DMM mice (Figure 2K). In contrast, OPN was rarely expressed overflowing the tidemark in the sham mice (Figure 2K).

These results show that the expression of *spp1* elevates earlier in IPFP than in the articular cartilage in DMM mice, and IPFP‐derived OPN may be accumulated at IPFP itself and the calcified cartilage.

### 2.3 IPFP‐Derived OPN Triggers OA Progression

**Figure 3:**
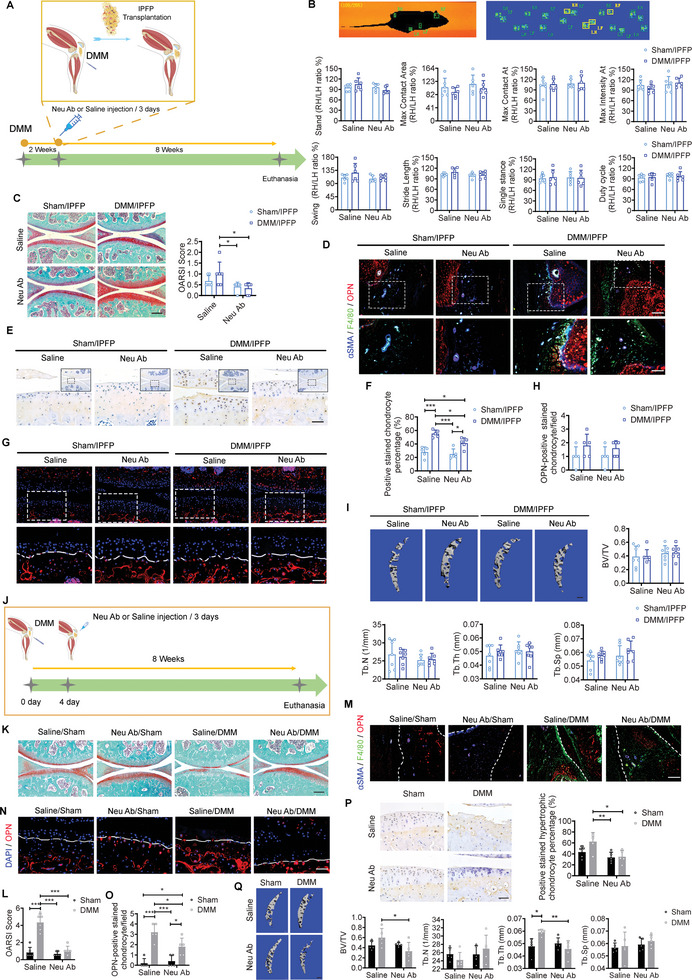
The role of IPFP‐derived OPN in OA progression. A) Schematic diagram illustrating the IPFP transplantation and treatment for the acceptor mice after IPFP transplantation. B) Images display pawprints of the left forelimb (LF), left hind limb (LH), right forelimb (RF), and right hind limb (RH) of a running mouse by automated gait analysis (Catwalk). Representative catwalk gait analysis among groups at week 8 post‐transplantation. *n* = 6 mice per group. C) Representative safranin O/fast green staining and quantification of OA severity by OARSI scores of medial tibial plateau among groups at week 8 post‐transplantation. *n* = 6 mice per group. Scale bar: 200 µm. D) Representative images of immunofluorescence staining of OPN, F4/80, and *α*SMA in IPFP among groups at week 8 post‐transplantation. Scale bar: 100 µm. The bottom row was magnified from the dotted frame in top row. Scale bar: 50 µm. E,F) Representative images (E) and quantification (F) of immunohistochemical staining of MMP9 in articular cartilage among groups at week 8 post‐transplantation. Scale bar: 50 µm. The image was magnified from the dotted frame part. *n* = 5 mice per group. Scale bar: 50 µm. G,H) Representative images (G) and quantification (H) of immunofluorescence staining of OPN in articular cartilage among groups at week 8 post‐transplantation. Scale bar: 100 µm. The bottom row was magnified from the dotted frame in top row. *n* = 5 mice per group. Scale bar: 50 µm. I) Representative µCT images and quantifications of subchondral trabecular bone among groups at week 8 post‐transplantation. *n* = 7 mice per group. Scale bar: 200 µm. J) Schematic diagram illustrating the saline or Neu Ab treatment for the sham or DMM mice without IPFP transplantation. K,L) Representative safranin O/fast green staining (K) and quantification (L) of OA severity by OARSI scores among groups at week 8 post‐surgery and injection. *n* = 6 mice per group. Scale bar: 200 µm. M) Representative images of immunofluorescence staining of OPN, F4/80, and *α*SMA in IPFP among groups at week 8 post‐surgery and injection. Scale bar: 100 µm. N,O) Representative images and quantification of immunofluorescence staining of OPN in articular cartilage among groups at week 8 post‐surgery and injection. *n* = 5 mice per group. Scale bar: 50 µm. P) Representative images and quantification of immunohistochemical staining of MMP9 in articular cartilage among groups at week 8 post‐surgery and injection. *n* = 5 mice per group. Scale bar: 50 µm. Q) Representative µCT images and quantifications of subchondral trabecular bone among groups at week 8 post‐surgery and injection. *n* = 5 mice per group. Scale bar: 200 µm. Images are representative of 3 independent experiments. All data are presented as mean ± SD. Neu Ab: OPN‐neutralizing antibody. Two‐way ANOVA with *Tukey's post hoc* test (B, C, F, H, I, L, O, P, and Q) were used. *p < 0.05, **p < 0.01, and ***p < 0.001. Parts of Figure 3A and 3J are created with BioRender.com.

To prove the roles of IPFP‐derived OPN participating in OA progression, we transplanted partial IPFP from the sham‐ or DMM‐mice to the compartment of IPFP in the normal mouse, whose articular cavity was simultaneously injected with saline or OPN‐neutralizing antibody (Neu Ab) every three days (**Figure** **3**A). The influence of transplantation on behaviors of mice was examined by the Cat‐walk apparatus (Figure 3B). The acquisition of stand, maximal contact area, maximal contact AT, maximal intensity AT, swing, stride length, single distance, and duty cycle were analyzed for detecting a subtle difference in locomotion. These measures were sensitive to disorders of mechanical stability posed by surgery treatments.^[^ ^35^ ^]^ Consequently, all parameters were comparable in the sham/IPFP and DMM/IPFP groups with or without Neu Ab treatment at week 8 post‐transplantation (Figure 3B). These results ruled out the functional limitations of IPFP transplantation produced on the recipient mice. Therefore, the graft combined with Neu Ab injection was designed to study the role of OPN in initializing the OA progression.

We observed less fibrillations in the DMM/IPFP group with Neu Ab treatment compared to the group with saline treatment (Figure 3C). OARSI score was compared among groups (Figure 3C). Neu Ab treatment substantially cleared the amount of free OPN and arrested *α*SMA expression in IPFP from the DMM/IPFP group as compared to the DMM/IPFP group (Figure 3D). Lots of MMP9‐positive stained chondrocytes appeared at the surface of articular cartilage in the DMM/IPFP group (Figure 3E,F). However, the number of OPN‐positive stained chondrocytes overflowing the tidemark was comparable among groups (Figure 3G,H). In consequence, subchondral trabecular bone mass was similar among groups no matter the treatment (Figure 3I). These data suggest that IPFP‐derived OPN triggers OA progression in terms of articular cartilage erosion.

### 2.4 Intra‐IPFP Injection of Neu Ab Ameliorates OA Progression

Since IPFP‐derived OPN is involved in the development of OA, it is possible to mitigate OA progression by reducing the concentration of OPN in the joint cavity (Figure 3J). DMM mice subjected to Neu Ab treatment for 8 weeks displayed remission of cartilage damage, such as aspects of cartilage erosion and matrix degradation quantified by OARSI grading (Figure 3K,L). The infiltrated macrophages and secreted OPN were significantly arrested in IPFP of the DMM group with Neu Ab treatment, and co‐localized OPN‐ and *α*SMA‐expressing areas markedly decreased over time (Figure 3M). The DMM mice treated with saline for 8 weeks exhibited typical hypertrophic phenotypes with significantly more MMP9‐positive stained hypertrophic chondrocytes and OPN‐positive stained chondrocytes overflowing the tidemark. In contrast, these features were blunted when the DMM mice received Neu Ab treatment for 8 weeks (Figure 3N–P). These findings implied the afflicted subchondral trabecular bone remodeling owing to the changes of chondrocyte hypertrophy and calcification in the deep chondrocytes, explaining that the DMM mice treated with Neu Ab displayed a significantly lower subchondral trabecular bone mass (BV/TV) than those received saline injection (Figure 3Q). These results suggest that Neu Ab injection confers cartilage protection from OA progression.

### 2.5 Dysregulated OPN/integrin *β*3 Signaling Involves in OA Established in both Mice and Patients

**Figure 4:**
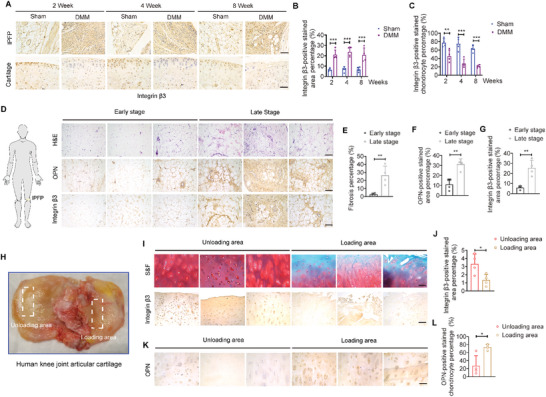
Dysregulated OPN and integrin *β*3 signaling in patients and mice. A–C) Representative images (A) and quantifications (B and C) of immunohistochemical staining of integrin *β*3 in IPFP (top row) and articular cartilage (bottom row) from sham and DMM mice at weeks 2, 4, and 8. *n* = 4 mice per group. Scale bar: 50 µm. D) Representative images of H&E staining and immunohistochemical staining of OPN (middle row) and integrin *β*3 (bottom row) in IPFP from ACLR patients at early‐stage (1‐2 months of injury period) and late‐stage (5‐6 months of injury period). Scale bar: 100 µm. E–G) Quantifications of H&E staining (E) and immunohistochemical staining of OPN (F) and integrin *β*3 (G) from (D). *n* = 4 patients per group. H) Human knee joint articular cartilages were obtained from total knee replacement of OA patients. White dashed boxes indicate respective loading and unloading areas. I) Representative safranin O/fast green (S&F) staining and immunohistochemical staining of integrin *β*3 in cartilage from TKA patients. Scale bar: 100 µm. J) Quantification of integrin *β*3‐positive stained area from (I). *n* = 4 patients per group. K,L) Representative images (K) and quantification (L) of immunohistochemical staining of OPN in deep cartilage from TKA patients. *n* = 4 patients per group. Scale bar: 100 µm. Images are representative of 3 independent experiments. All data are presented as mean ± SD. Two‐way ANOVA with *Sidak's post hoc* test (B and C) and two‐tailed *Welch's t*‐test (E, F, G, J, and L) were used. *p < 0.05, **p < 0.01, and ***p < 0.001.

Integrin *β*3, one of OPN receptors, is involved in synovial cell proliferation, differentiation, and migration.^[^ ^36^ ^]^ IHC staining confirmed that integrin *β*3 was enriched in IPFP of the DMM mice, especially in tubular structures (**Figure** **4**A,B), echoing to the site where was abundant with *α*SMA‐ and OPN‐expression. Besides, integrin *β*3 was significantly inhibited in articular cartilage surface (Figure 4A–C), where was also absent with OPN expression. Histological analysis of the IPFP from ACL reconstruction (ACLR) patients also displayed severe fibrosis in late‐stage relative to the early‐stage (Figure 4D,E). Both expression levels of OPN and integrin *β*3 were significantly higher in late‐stage than in early‐stage (Figure 4D,F,G). The infiltrated macrophages in IPFP of the late‐stage were significantly higher than that in early‐stage (Figure S2, Supporting Information). These findings suggest that the increased expression of integrin *β*3 may enable the infiltrated cells within IPFP to elicit pathological responses, and thereby drive the fibrosis progression. Besides, integrin *β*3 was almost blunted in the articular cartilage of the loading area from OA patients relative to the unloading area (Figure 4H–J), while the cartilage around tidemark under the loading area from OA patients displayed a higher percentage of OPN‐positive stained chondrocytes relative to that under the unloading area (Figure 4K,L), well in line with our results found in mice.

The circulatory system can buffer local fluctuations of OPN levels,^[^ ^19^ ^]^ thus high dose and frequency of intra‐IPFP injection of Neu Ab is essential for reviving OPN level in joint. Above studies either from mouse or human suggested that endogenous integrin *β*3 (*Cd61*) was abundant for OPN functions that triggered the OA progression. Besides, Itgb3^−/−^ (Integrin *β*3 deficient) mice show markedly attenuated cartilage degeneration and synovitis as compared to Itgb3^+/+^ mice at week 20 post‐DMM surgery,^[^ ^25^ ^]^ providing a rationale for disease‐modifying therapy by targeting integrin *β*3. Hence, we would develop a nanogel delivery system to prolong the retention and efficiency of siRNA *Cd61* with RGD sequence in the joint cavity.

### 2.6 Synthesis and In Vitro Characterization of ^RGD−^Nanogel/siRNA *Cd61*

**Figure 5:**
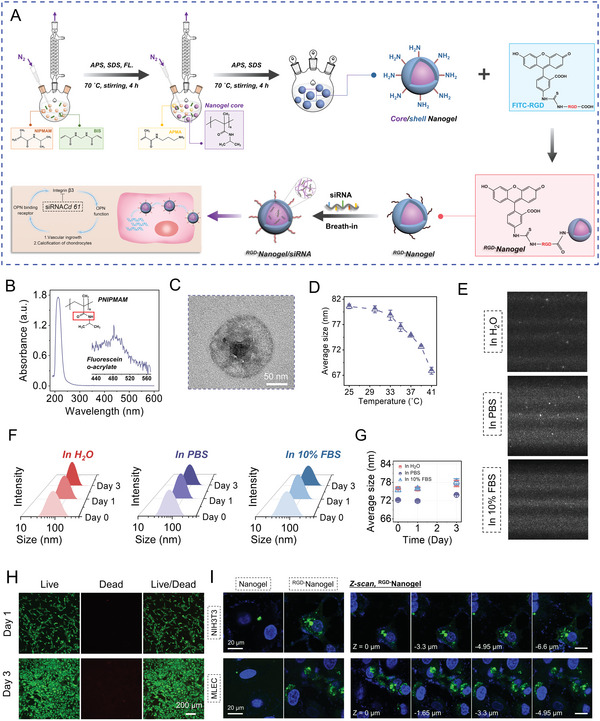
Synthesis and properties of ^RGD−^Nanogel/siRNA *Cd61*. A) Illustration of ^RGD−^Nanogel/siRNA *Cd61* synthesis and function. APS: ammonium persulfate; SDS: sodium dodecyl sulfate; FL.: fluorescein O‐acrylate. B) UV–vis spectrum of the synthesized PNIPMAM nanogel. C) The representative images of the core/shell nanogel particle by transmission electron microscopy (TEM). D) The analysis of average size of nanogel in deionized water (D. I. H_2_O) under different temperatures by dynamic light scattering (DLS). *n* = 3 per group. E) The video frames of nanogel particles in D. I. H_2_O, PBS, and 10% fetal bovine serum (FBS) at 37 ˚C, obtained from nanoparticle tracking analysis (NTA) measurement. F) Representative curves of the size distribution of the nanogel particles in D. I. H_2_O, PBS, and 10% FBS at 37 ˚C from day 0 to day 3. G) Evolution of the average size of the nanogel particles in different aqueous environments from day 0 to day 3, obtained from DLS measurements. *n* = 3 per group. H) Representative live/dead cell staining images. I) Fluorescent confocal images of 3T3 fibroblasts (NIH3T3) and murine lung endothelial cells (MLEC) incubated with nanogel or ^RGD−^nanogel particles for 6 hours, where the nanogel or ^RGD−^nanogel particles utilized did not contain siRNA *Cd61*. The cell nuclei were stained with DAPI. Confocal microscopy *Z*‐scan was further performed on the cells incubated with ^RGD−^nanogel particles from the top to bottom of cells with a step size of 0.33 µm. Images are representative of 3 independent experiments.

The core/shell nanogels were synthesized by two‐step precipitation and possessed a strong absorption peak at 213 nm based on the multiple amide bonds in poly(*N*‐isopropylmethacrylamide) (PNIPMAM) (**Figure** **5**A,B). The effective integration of the fluorescein O‐acrylate (FL.) dye in the nanogel core was confirmed by the slight absorbance at around 490 nm (Figure 5B). Transmission electron microscopy (TEM) scanning of nanogel displayed a loose shell (light color) outside the dense core (dark color) by different crosslinking density (Figure 5C). Dynamic light scattering (DLS) analysis displayed a slight shrinkage of average nanogel size from 80.58 ± 0.44 to 67.90 ± 0.70 nm ranging from 25 to 41 ˚C (Figure 5D). Besides, the critical point (from swollen to collapse) of the nanogel was around 43 ˚C,^[^ ^37^ ^]^ and there was only a ≈4 nm variation of particle volume from 35 to 39 ˚C, indicating the satisfactory stability under the physiological temperature (37 ˚C) (Figure 5D). To further confirm the long‐term structural stability of the nanogel for manifold settings, we measured the particle size distributions in deionized water (D. I. H_2_O), phosphate buffer saline (PBS), and 10% fetal bovine serum (FBS) at 37 ˚C, respectively. Three cohorts showed comparable size distribution, with an ignorable increase of the average particle size (≈2 nm) from day 0 to day 3 (Figure 5E–G), indicating the homogeneous nanogel suspension without aggregation. Live/Dead cell staining showed that the concentration of nanogel (250 µg mL^−1^) used did not generate any negative effect to the cell survival (Figure 5H).

To test the RGD‐anchoring‐dependent attachment, we performed a cellular uptake experiment to ^RGD−^Nanogel (without siRNA *Cd61*) with 3T3 fibroblasts and murine lung endothelial cells (MLEC). Both cohort experiments showed substantial ^RGD−^Nanogel particles were captured inside cells relative to nanogel group after incubation for 6 hours, indicating the increased endocytosis in ^RGD−^Nanogel group (Figure 5I).

### 2.7 The IPFP‐Targeting Efficiency of ^RGD−^Nanogel and Transfection Efficiency of ^RGD−^Nanogel/siRNA *Cd61* In Vivo

**Figure 6:**
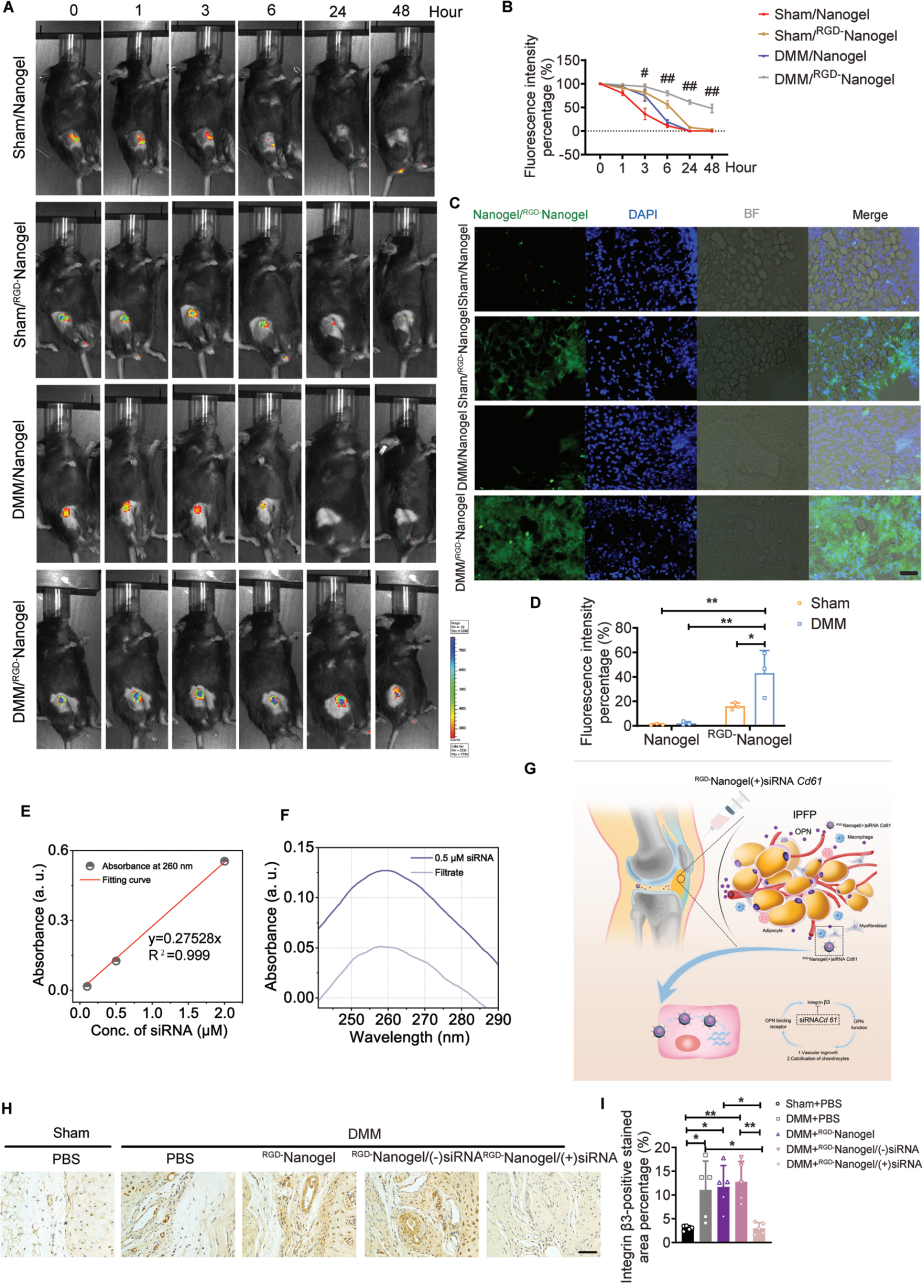
The IPFP‐targeting efficiency of ^RGD−^Nanogel and transfection efficiency of ^RGD−^Nanogel/siRNA *Cd61* in vivo. A) The IPFP‐targeting efficiency of Nanogel or ^RGD−^Nanogel was visualized by IVIS system at 0‐, 1‐, 3‐, 6‐, 24‐, and 48‐hours post‐injection. B) The value was calculated by the quantified fluorescence intensity relative to the initial fluorescence intensity. #, *p* < 0.05 between DMM/Nanogel group and DMM/^RGD−^Nanogel group. ##, *p* < 0.0001 between DMM/Nanogel group and DMM/^RGD−^Nanogel group. *n* = 3 mice per group. C) The IPFP‐targeting efficiency of Nanogel or ^RGD−^Nanogel was evaluated by IPFP cryosection on day 2 post‐injection. Scale bar: 50 µm. D) Quantifications of fluorescence intensity of Nanogel or ^RGD−^Nanogel in IPFP cryosection from (C). *n* = 3 mice per group. E) Standard curve of the absorbance (at 260 nm) versus siRNA concentration. F) UV–vis spectra of the siRNA loading solution and the filtrate of siRNA‐loaded nanogel suspension. G) ^RGD−^Nanogel/siRNA *Cd61* was weekly intra‐IPFP injected for investigation of its pharmacodynamics in OA mice. H,I) Representative images (H) and quantification (I) of immunohistochemical staining of integrin *β*3 in IPFP among groups at week 8 post‐surgery. *n* = 5 mice per group. Scale bar: 50 µm. Images are representative of 3 independent experiments. All data are presented as mean ± SD. Two‐way ANOVA with *Sidak's post hoc* test (B), two‐way ANOVA with *Tukey's post hoc* test (D), and one‐way ANOVA with *Tukey's post hoc* test (I) were used. *p < 0.05 and **p < 0.01.

The in vivo activities of ^RGD−^Nanogel were monitored in sham and DMM mice post‐injection. First, the IPFP‐targeting efficiency of Nanogel or ^RGD−^Nanogel was visualized and evaluated by the IVIS imaging system in sham and DMM mice at week 2 post‐surgery. RGD peptides modified nanogel particles gained longer lasting‐anchor on the IPFP as compared to the nanogel group, while the fibrosis of IPFP in DMM group rendered the significantly increased retention of ^RGD−^Nanogel than that in other groups (**Figure** **6**A,B). Moreover, cryosection of IPFP treated with nanogel or ^RGD−^Nanogel was scanned and digitalized with a microscopic imaging system (Figure 6C). DMM/^RGD−^Nanogel group displayed the greatest fluorescence intensity among groups on day 2 post‐injection (Figure 6D).

The siRNA *Cd61* molecules were embedded into the freeze‐dried nanogel particles through a well‐established “breathing‐in” strategy, with a relatively high encapsulation efficiency (EE) of ≈60.6% (Figure 6E,F). To further investigate the effect of ^RGD−^Nanogel/siRNA *Cd61* on OA progression, we weekly injected ^RGD−^Nanogel/siRNA *Cd61* into the articular cavity starting at day 8 post‐surgery (Figure 6G). ^RGD−^Nanogel/siRNA *Cd61* substantially obviated the expression of integrin *β*3 in IPFP of the DMM mice at week 8 post‐surgery (Figure 6H,I). Hence, the ^RGD−^Nanogel/siRNA *Cd61* possesses a lasting IPFP‐targeting efficiency and a desired transfection efficiency in vivo.

### 2.8 Intra‐IPFP Injection of ^RGD−^Nanogel/siRNA *Cd61* Mitigates the OA Progression in Mice

**Figure 7:**
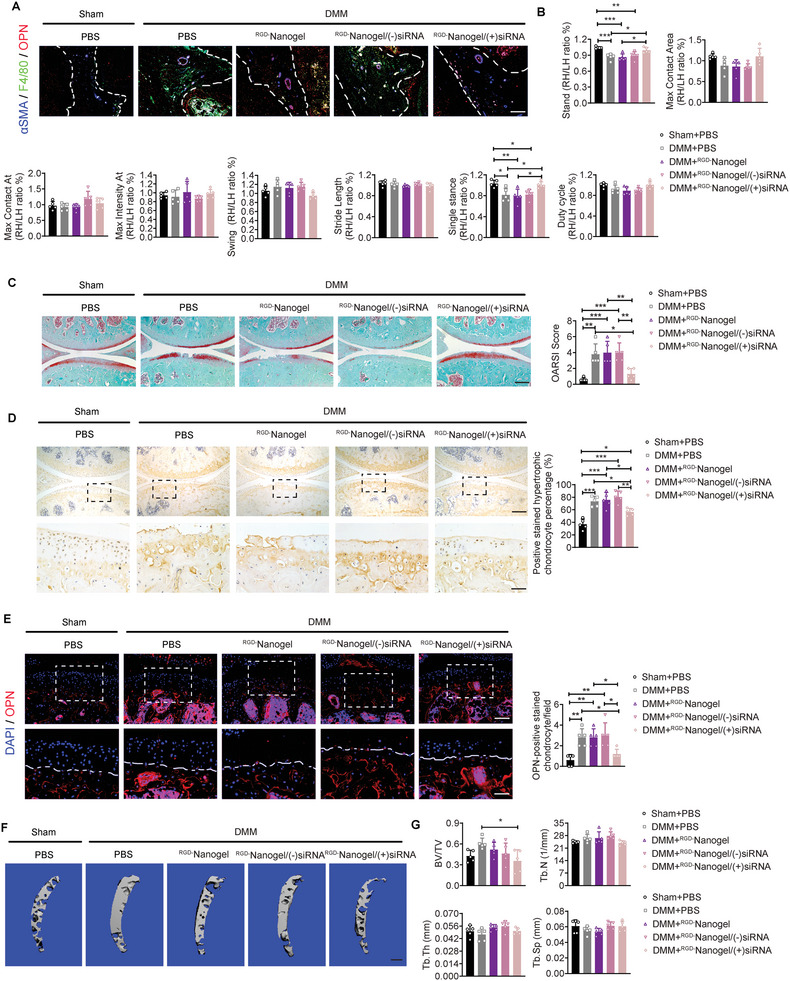
Intra‐IPFP injection of ^RGD−^Nanogel/siRNA *Cd61* to mitigate the OA progression. A) Representative images of immunofluorescence staining of OPN, F4/80, and *α*SMA in IPFP among groups at week 8 post‐surgery and injection. Scale bar: 100 µm. B) Representative catwalk gait analysis among groups at week 8 post‐surgery and injection. *n* = 5 mice per group. C) Representative safranin O/fast green staining and quantification of OA severity by OARSI scores of knee joint sections among groups at week 8 post‐surgery and injection. *n* = 5 mice per group. Scale bar: 200 µm. D) Representative images and quantification of immunohistochemical staining of MMP9 in articular cartilage among groups at week 8 post‐surgery and injection. Scale bar: 200 µm. The bottom row was magnified from the dotted frame in top row. *n* = 5 mice per group. Scale bar: 50 µm. E) Representative images and quantification of immunofluorescence staining of OPN in articular cartilage among groups at week 8 post‐surgery and injection. Scale bar: 100 µm. The bottom row was magnified from the dotted frame in top row. *n* = 5 mice per group. Scale bar: 50 µm. F,G) Representative µCT images (F) and quantifications (G) of subchondral trabecular bone among groups at week 8 post‐surgery and injection. *n* = 5 mice per group. Scale bar: 200 µm. Images are representative of 3 independent experiments. All data are presented as mean ± SD. One‐way ANOVA with *Tukey's post hoc* test (B, C, D, E, and G) were used. *p < 0.05, **p < 0.01, and ***p < 0.001.

**Figure 8:**
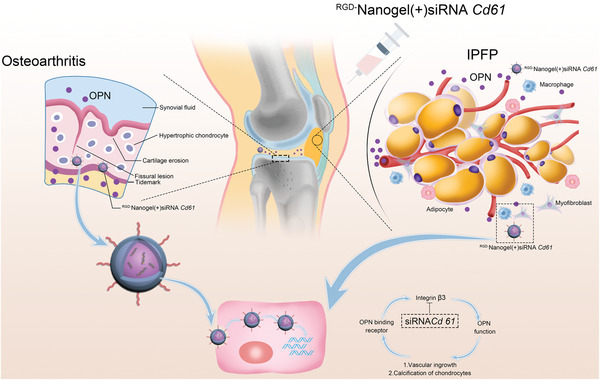
Schematic diagram of siRNA‐releasing nanogel for OA treatment by disrupting the signaling of infrapatellar fat pad‐derived OPN.

The DMM mice subjected to ^RGD−^Nanogel/siRNA *Cd61* exhibited less more infiltrated macrophages and co‐localized OPN‐ and *α*SMA‐expressing area in IPFP relative to the DMM mice treated with PBS (**Figure** **7**A). Although partial parameters from the Cat‐walk apparatus were comparable in different groups, the acquisition of stand and single stance indicated that ^RGD−^Nanogel/siRNA *Cd61* treatment imparted the DMM mice functional behaviors similar to the sham mice (Figure 7B). Safranin O/fast green staining quantified by OARSI score displayed mitigated cartilage damage in the mice treated with ^RGD−^Nanogel/siRNA *Cd61* compared to other treatments in the DMM mice (Figure 7C). Both numbers of MMP9‐positive stained hypertrophic chondrocytes in calcified cartilage and OPN‐positive stained chondrocytes overflowing the tidemark were lower in the mice treated with ^RGD−^Nanogel/siRNA *Cd61* than other treatments (Figure 7D,E). Moreover, the subchondral trabecular bone mass (BV/TV) was also significantly lower in the mice treated with ^RGD−^Nanogel/siRNA *Cd61* than that in the DMM mice with other treatments (Figure 7F,G). Token together, these results demonstrate that intra‐IPFP injection of ^RGD−^Nanogel/siRNA *Cd61* biologically targets and inhibits the expression of Cd*61* in the joint cavity, thus attenuating OA progression by dampening OPN functions (**Figure** **8**).

## 3 Discussion

This study demonstrated a previously unidentified function of IPFP‐derived OPN that serves as a determinant in OA progression, and inhibition of the OPN may be an effective strategy for OA treatment. The current study has provided mechanistic insights into the complex factors that underlie OA pathophysiology and offers prospects for alleviating the damaged cartilage. Briefly, IPFP‐derived OPN participates in OA progression, including chondrocyte hypertrophy and IPFP fibrosis, and intra‐IPFP injection of tailored ^RGD−^Nanogel/siRNA *Cd61* effectively arrests the cartilage erosion and IPFP inflammation, suppresses the advancement of tidemark, and reduces the subchondral trabecular bone mass, leading to an innovative therapeutic strategy to mitigate OA progression.

To avoid damaging the IPFP in surgery, all intra‐articular manipulations for the DMM model are conducted under a stereo zoom microscope. Even then, we also observe rapidly deteriorated phenotypes in IPFP, including the disappearance of adipocytes, fibrosis, and immune cells infiltration. Comparatively, monoiodoacetic acid (MIA)‐induced rat joint inflammation model and post‐traumatic osteoarthritis (PTOA) model, both non‐invasive manners for OA also display marked inflammation in IPFP as that in the DMM model.^[^ ^14^, ^38^, ^39^ ^]^ Of note, age may also affect the morphometry and content of IPFP in case of no pathological condition, indicating a strong evidence that IPFP activation may contribute to the knee joint diseases, such as OA.^[^ ^40^ ^]^ These results suggest that IPFP is much more sensitive to inflammation with features such as infiltration of immune cells, necrosis of adipocytes, and formation of fibrosis.

Cytokines and adipokines are mainly found in the synovial fluid of OA patients,^[^ ^10^, ^41^ ^]^ and IPFP‐derived media shows a trend toward more IL‐6, adiponectin, and adipsin,^[^ ^15^ ^]^ indicating that IPFP may be a main source and an endocrine organ. We found that IPFP was involved in the cascade amplification of inflammation by secretory cytokines, even though IPFP was not the initial injury site in DMM mice. Indeed, the inflammatory responses closely rely on rich vascular and neural supplies from IPFP.^[^ ^42^ ^]^ The activated immune cells may interact with adipocytes and periarticular tissues. Then crown‐like structure (CLS) in the stromal vascular fraction of adipose tissue arises to clear the necrotic adipocytes and other cells. During this process, alternating CLS formation and chronic inflammatory responses promote fibrosis and cytokines secretion.^[^ ^19^ ^]^ As a matricellular phosphoglycoprotein, OPN executes as an adaptor and modulator of cell‐matrix interactions via binding to integrins. Hence, OPN regulates the metabolic pathophysiology of IPFP via an autocrine pathway.

Our spatiotemporal analysis of the articular cartilage reveals the early occurrence of fibrosis in IPFP and advanced osteoarthritic changes, including fissuring and fragmentation of the cartilage, chondrocytes hypertrophy, advancement of the tidemark, expansion of the calcified cartilage area, and thickening of the subchondral trabecular bone. The chondrocytes exhibit low mitotic activity and minimal collagen turnover under physiological conditions, but the cartilage matrix displays striking alterations in its composition under OA conditions.^[^ ^1^, ^43^ ^]^ The disruption of the pericellular matrix exposing to chondrocytes deregulates the function of chondrocytes through their surface receptors, including integrins.^[^ ^44^ ^]^ When the key component of integrin *α*v*β*3 is specifically inhibited, the DMM mice display attenuation of OA phenotypes, including mitigated inflammation and joint destruction.^[^ ^25^ ^]^ As the loss of the progressive proteoglycans followed by the erosion of collagen network, several different matrix metalloproteinases (MMPs) execute functions.^[^ ^45^, ^46^ ^]^ MMP9 plays key roles in angiogenesis of growth plate and apoptosis of hypertrophic chondrocytes in developmental mice, and mutation of MMP9 results in the delayed growth plate remodeling and conversion of chondrocytes into osteoblasts in zebrafish.^[^ ^34^, ^47^ ^]^ Further, the increased presence of MMP9 in synovial fluid of patients with OA compared with the healthy controls suggests that MMP9 is likely to be involved in the degradation of articular collagen.^[^ ^48^, ^49^ ^]^ Our current study directly observes the increased expression of MMP9 in osteoarthritic cartilage, especially in the calcified cartilage area, where might be essential for chondrocytes hypertrophy and calcification depending on OPN/*α*v*β*3 complexes. Therefore, IPFP‐derived OPN contributes to the processes of hypertrophy and ossification of chondrocytes through a paracrine pathway.

Current pharmacological treatments are mostly inclined to relieve symptoms, but not for recusing or preventing the OA progression.^[^ ^50^ ^]^ Since the unsatisfactory efficiency of pharmacological treatment for OA, substantial efforts have gone into the development of biological drugs and therapies.^[^ ^51^, ^52^, ^53^ ^]^ The sustainable and high‐efficiency agents have been the most crucial considerations to attain a long‐term effect on OA treatment, thus gene therapy may be the appreciated approach. Adeno‐associated virus (AAV) gene therapy has been limited due to immunogenicity and random integration, indicating that AAV therapy has a long journey to unlock the full potential for in vivo gene therapy.^[^ ^54^ ^]^ Hence, the use of nonviral vectors for gene therapy has relative advantages in terms of safety compared with the viral method. Among potential therapeutic alternatives, the siRNA‐based intervention has a great expectation as an innovative therapy applied for OA treatment. The systemic approach of siRNA is preferential to treat inflamed joints of the whole body in rheumatic diseases, and local injection of siRNA is favored for a single afflicted joint to rescue cartilage destruction.^[^ ^26^ ^]^ Conventional intra‐articular injection of siRNA urgently requires an approach to overcome rapid clearance and physiological obstacles.^[^ ^55^ ^]^ Established formulations such as hydrogels, suspensions, lipid‐based formulations, and nano‐particles currently sprout and arborize.^[^ ^56^ ^]^ The formulations combined with siRNA for OA therapy offer a promising manner to solve these nuisance problems. As siRNA is hydrophilic molecules that are unable to combine and integrate into the cell membrane, where will be rapidly degraded by endogenous enzymes in vivo.^[^ ^57^ ^]^ Nanogel has a high degree of porosity that possesses the ability to encapsulate macromolecular, while the high moisture content endows its biocompatibility and stimuli‐responsive polymers yield colloids that are responsive to their local environment.^[^ ^57^ ^]^ Both articular cartilage and IPFP express OPN and its ligands. Above results indicate that endogenous integrin *β*3 is sufficient in inflammatory IPFP to trigger the OA progression. Hence, there is positive feedback between IPFP‐derived OPN and fibrosis in IPFP that can be interrupted by blocking either integrin *β*3 or OPN. The nanogel loaded with siRNA *Cd61* and conjugated with RGD, conserved motif in OPN, mostly binds at a site with higher expression of integrin *β*3. Therefore, ^RGD−^Nanogel/siRNA *Cd61* silences the transcription of *Cd61* to prevent IPFP from progressive fibrosis and macrophages infiltration. Importantly, ^RGD−^Nanogel/siRNA *Cd61* approach considers the early alterations of IPFP and cartilage before occurring structural and functional anomalies in OA, suggesting a great potential for developing novel and targeted therapies for clinical application.

Additional concerns of investigations are unmet needs for the interpretation of our findings. First, the IPFP‐derived OPN shows a profound influence on OA progression, but the initial factors driving the homeostasis disorders of IPFP remain elusive. Besides, the present study also does not eliminate the possibility that OPN from other sources of tissues (such as synovium and subchondral bone) or other cytokines (such as MMP13 and Il‐1*β*) from IPFP playing roles in OA progression. In addition, we have mainly shown the relevance among the expression of OPN or integrin *β*3 in specimens from patients instead of the definitive clarification as shown in OA mice. As it was not feasible for us to harvest the degenerative specimens at the onset of IPFP in aging patients, alternatively we observed IPFP pathological changes with OPN/integrins expression using ACLR patients, albeit IPFP characteristics of late‐stage ACLR patients were different as to compare with those of OA patients.^[^ ^58^ ^]^ Besides, as no cartilage from young ACLR patients is available for comparison, we collected cartilage from total knee arthroplasty patients to study OPN/integrins expression in OA progression. Lastly, although ^RGD−^Nanogel/siRNA *Cd61* approach mostly integrates and inhibits the sites of lesion expressing *Cd61*, the physiological requirement for integrins in the normal area might be affected.

## 4 Conclusion

Our investigation demonstrates that IPFP aberration‐associated OPN plays a crucial role in OA progression, including articular chondrocyte hypertrophy and IPFP fibrosis, and reduction of OPN by intra‐IPFP injection of Neu Ab obviates the joint degeneration by limiting the functionalized MMP9 and *α*SMA. The ^RGD−^nanogel particles designed in this study effectively attenuate the cartilage erosion and IPFP inflammation, suppress the advancement of tidemark, and reduce the subchondral trabecular bone mass, leading to a development of an innovative therapeutic strategy to mitigate OA progression. Our proof‐of‐concept study suggests that the reduction of OPN secretion possesses a potential for OA treatment and the tailored ^RGD−^Nanogel/siRNA method offers an avenue for the development of an innovative therapeutic strategy.

## 5 Experimental Section

### Animals

Twelve‐week‐old male *C57BL/6* mice were subjected to destabilization of the medial meniscus (DMM) surgery or sham surgery at the right knee as described previously.^[^ ^59^ ^]^ Briefly, for the DMM surgery, the skin and joint capsule of mice were opened after anesthesia with ketamine (60 mg kg^−1^, i.p.) and xylazine (4 mg kg^−1^, i.p.). Microscopic instruments were used to access the joint space from the less fatty area of the IPFP (Figure S3, Supporting Information), and the medial meniscotibial ligament was cut to destabilize the meniscus, which was free to displace medially. To avoid damaging the IPFP, all intra‐articular manipulations were done under a stereo zoom microscope (Bresser, 58–03100 Stereo Microscope). For the sham surgery, the skin and joint cavity were opened in the same procedures without any further damage. The incision of the joint capsule and skin would be closed in layers with 7‐0 prolene sutures and 5‐0 vicryl sutures. All procedures were performed under aseptic. Mice were humanely euthanized when they appeared suffering during the observation period. All mice were kept at the Experimental Animal Center at the Prince of Wales Hospital in Hong Kong under a 12‐hour light/dark cycle, 17–24 °C ambient temperature, 70% humidity, and received food and water ad libitum. Mice were sacrificed by intraperitoneal injection of an overdose of sodium pentobarbital. The specified experimental protocols were approved by the Animal Experiment Ethics Committee of the Chinese University of Hong Kong (21‐021‐MIS and 22‐089‐MIS).

### Human Samples

After obtaining approval from the Joint Chinese University of Hong Kong‐New Territories East Cluster Clinical Research Ethics Committee (CUHK‐NTEC CREC, reference number: 2013.248), the tibial plateau was collected from total knee arthroplasty (TKA) patients (5 male patients, 60–80 years old). The demographic information of patients was collected. The lateral tibial plateau (LTP) was regarded as the unloading area, and the medial tibial plateau was regarded as the loading area. Specimens were prepared for histological analysis after decalcification.

After approval by the Joint Chinese University of Hong Kong‐New Territories East Cluster Clinical Research Ethics Committee (CUHK‐NTEC CREC, reference number: 2018.109), IPFP was collected from patients with anterior cruciate ligament reconstruction (ACLR, 5 male patients with 1–2 months of injury period (early‐stage, comparable with the early‐stage of IPFP fibrosis in mouse DMM), 23–35 years old; 5 male patients with 5–6 months of injury period (late‐stage, comparable with the late‐stage of IPFP fibrosis in mouse DMM), 18–30 years old). The demographic information of patients was collected. Specimens were prepared for histological analysis.

### Histological Analysis

The isolated knee joints were fixed in 4% paraformaldehyde (PFA, Thermo Fisher Scientific, USA, cat# A11313) for 24 h and decalcified in 12.5% ethylenediaminetetraacetic acid (EDTA, pH 7.4, Sigma, USA, cat# 798681) for 14 days. EDTA solution was changed every three days. Then samples were embedded in paraffin and sectioned to 5 µm thickness. Sections were stained with safranin O/fast green (Sigma, USA, cat# S8884/F7258) for histologic scoring OA based on Osteoarthritis Research Society International (OARSI) assessment criteria.^[^ ^60^ ^]^ Sections were stained according to a routine Picro‐Sirius red (Abcam, UK, cat# ab150681) staining protocol for quantifying the collagen contents. Haematoxylin and Eosin (H&E, Solarbio, China, cat# G1120) staining was performed for evaluating the synovitis according to the previous description.^[^ ^61^ ^]^ Sections stained with H&E were used for the quantification of fibrosis.^[^ ^12^, ^62^ ^]^ Three sections, echoing to 25%, 50%, and 75% points of an entire IPFP, of each sample were captured digital images and quantified by ImageJ software (version 1.52v, USA) with color thresholds. Each quantification subtracted the vasculature area that was manually quantified by ImageJ software. The quantification of total fibrosis in one sample was calculated by the average of three sections. To measure the thickness of articular cartilage, three sections, echoing to 25%, 50%, and 75% points of the tibial plateau from the medial collateral ligament side to medial intercondylar nodes, of each sample were stained with safranin O/fast green and quantified by ImageJ software (version 1.52v, USA) (Figure S4, Supporting Information).^[^ ^59^ ^]^

### Immunohistochemical and Immunofluorescence Staining

For immunohistochemical staining, sections were deparaffinized by xylene and hydrated by gradient alcohols.^[^ ^19^ ^]^ After treatment with 3% hydrogen peroxide for 15 min in dark, sections were immersed in sodium citrate buffer (10 mm sodium citrate, 0.05% Tween 20, pH 6.0) for 30 min at 80 °C. After that, sections were permeabilized with blocking buffer (1% bovine serum albumin (BSA, Sigma, USA, cat# A7906) and 0.1% Triton X‐100 in PBS) for 30 min at room temperature. Primary antibodies were incubated that recognized OPN (Abcam, UK, cat# ab8448, 1:100), MMP9 (Abclonal, USA, cat# A2095, 1:100), Integrin *β*3 (Thermo Fisher Scientific, USA, cat # PA585926, 1:100), and COL1A1 (Abclonal, USA, cat# A16891, 1:50) diluted in 1% BSA buffer (1% BSA in PBS) overnight at 4 °C. Subsequently, sections were incubated with HRP‐labelled secondary antibody (Abcam, UK, cat# ab6721,1:200) diluted in 1% BSA for 2 h at room temperature. The 3,3′‐Diaminobenzidine (DAB, Thermo Fisher Scientific, USA, cat# 34002) was used for color development and hematoxylin was used for nucleus counterstain. Sections were omitted the primary antibody but incubated with secondary antibody as negative controls.

For immunofluorescence staining,^[^ ^19^ ^]^ after deparaffinization and rehydration, sections were subjected to a sodium citrate buffer for 30 min at 80 °C. Sections were permeabilized with blocking buffer (1% BSA and 0.1% Triton X‐100 in PBS) for 30 min at room temperature. Primary antibodies were incubated that recognized OPN (Invitrogen, USA, cat# PA125152, 1:100), F4/80 (Abcam, UK, cat# ab6640, 1:200), and *α*SMA (Abcam, UK, cat# ab124964, 1:200) diluted in 1% BSA buffer overnight at 4 °C. Species‐matched secondary antibodies, Alexa Fluor 405 (Abcam, UK, cat# ab175651, 1:300), Alexa Fluor 488 (Thermo Fisher Scientific, USA, cat# A48262, 1:200), and Alexa Fluor 546 (Thermo Fisher Scientific, USA, cat# A‐11056, 1:200), were used for 1 h at 37 °C in the dark. The sections were mounted with DAPI (Thermo Fisher Scientific, USA, cat# P36931). Sections were omitted the primary antibody but incubated with secondary antibody as negative controls. Sections were scanned and digitalized with a microscopic imaging system (Leica DM5500; Leica Micro‐systems, Wetzlar, Germany).

### Micro‐Computed Tomography (µCT) Imaging

The knee joints of mice were scanned by a µCT‐40 imaging system (Scanco Medical, Brüttisellen, Switzerland) with a resolution of 10 µm per pixel as per a previous description.^[^ ^63^ ^]^ The scanner was set at a voltage of 70 kVp, and a current of 114 µA. Sagittal images of the tibiae subchondral bone were used to perform 3D histomorphometric analysis. The tibial plateau was artificially divided into four equal parts from medial to lateral. The region of interest (ROI) was defined to cover 16 slices of the subchondral trabecular bone between subchondral cortical bone and growth plate at the quarter‐point of the tibial plateau from the medial collateral ligament side (Figure S5, Supporting Information). The Gaussian filter with Sigma = 0.8, Support = 1, and threshold = 287 HU were used to reconstruct 3D images. The analysis of microstructural parameters covered bone volume fraction (BV/TV), trabecular bone thickness (Tb.Th), trabecular bone separation (Tb.Sp), and trabecular bone number (Tb.N).

### Conditioned Medium

Mice were sacrificed to collect the IPFP at weeks 2, 4, and 8 post‐DMM or ‐sham surgery. All procedures were performed under aseptic conditions. The IPFP from each mouse was cultured in 0.6 mL FBS‐free *α*‐Minimum Essential Medium (*α*‐MEM, Thermo Fisher Scientific, USA, cat# 11900024) containing 1% penicillin‐streptomycin‐neomycin (PSN, Gibco, USA, cat# 15640055) for 3 days at 37 °C and 5% CO_2_. The conditioned medium was collected and centrifuged at 500 g for 5 min, then the supernatant was kept at −80 °C until assayed.

### Enzyme‐Linked Immunosorbent Assay (ELISA)

The concentration of IPFP‐secreted OPN in the conditioned medium was measured by ELISA (Abcam, UK, cat# ab100734) as protocol described. Briefly, the stored supernatant was diluted for 25‐fold using the assay buffer. The optical density of each well was immediately measured using a microplate reader (BioTek Quant Microplate Spectrophotometer, USA) at 450 nm.

### IPFP Transplantation

The fat pad was transplanted as a previous description.^[^ ^64^ ^]^ To avoid immunological rejection, donor IPFP was acquired from littermates. IPFP‐donor individuals, referring to the mice whose IPFP was reserved for transplantation, were euthanasia at week 2 post‐sham or ‐DMM surgery. Acceptor mice, 14‐week‐old male *C57BL/6* mice, were anesthesia with ketamine (60 mg kg^−1^, i.p.) and xylazine (4 mg kg^−1^, i.p.). The donated IPFP was implanted into the original intercompartment of IPFP in acceptor mice whose medial meniscus was kept intact without any surgery. The incision of the joint capsule and skin would be closed in layers with 7‐0 prolene sutures and 5‐0 vicryl sutures. To avoid damaging the IPFP of acceptor mice, all intra‐articular manipulations were done under a stereo‐zoom microscope. All procedures were performed under aseptic. The mouse was housed individually for one week after surgery and then evaluated the gait abnormality by Catwalk XT 9.0 system (Noldus, Wageningen, Netherlands). Mice were sacrificed at week 8 post‐transplantation.

### Gait Analysis

Gait analysis was performed by the Catwalk XT 9.0 system (Noldus Information Technology, Wageningen, the Netherlands) to evaluate gait abnormality according to the protocol.^[^ ^65^ ^]^ Briefly, each mouse was trained to get familiar with the Catwalk system before the formal experiment to walk ad libitum across the glass walkway (width, 5 cm) for image picking. Records of paw prints were automatically triggered when the mice entered the ROI. Pawprints were classified for left forelimb, right forelimb, left hind limb, and right hind limb by the built‐in software. Each mouse was successfully recorded at least three times and any record included about an average number of four cycles of complete steps. Successful records for one crossing were straight without any interruption or hesitation and allowed a maximum 30% speed variation. The records with normal footfall patterns were further analyzed after manually checking the correctness of the classification. The acquisition of stand, maximal contact area, maximal contact AT, maximal intensity AT, swing, stride length, single distance, and duty cycle were collected and analyzed for comparison among groups.

### Neu Ab Injection

OPN‐neutralizing antibody (Neu Ab, Thermo Fisher Scientific, USA, cat# PA1‐25152) injection for the cohorts of IPFP transplantation or DMM treatment, anesthesia was maintained by mask inhalation of isoflurane vaporized at concentrations of up to 4% in the induction phase and 0.8‐1.3% during prolonged experimental observations. The knee joint hair of the mice was gently shaved, and the skin was treated with betadine. OPN was depleted by locally injecting Neu Ab (0.1 µg/unilateral/3 days) into the subcapsular IPFP without a surgical incision. The control mice received a matched injection of saline (15 µL/unilateral/3 days). All mice were returned to the cages when they were awake. Mice will be sacrificed by intraperitoneal injection of an overdose of sodium pentobarbital at week 8 post‐transplantation or DMM surgery. The knee joint was collected for histological and biological analysis.

### Synthesis of Core/Shell poly (*N*‐isopropylmethacrylamide) (PNIPMAM) Nanogel

The core/shell PNIPMAM nanogel was fabricated according to the established protocol.^[^ ^57^, ^66^ ^]^ First, the nanogel core particles were synthesized via a free‐radical precipitation polymerization. The molar ratio of the monomer, *N*‐isopropylmethacrylamide (NIPMAM, Sigma, USA, cat# 423548) and the crosslinker, *N,N’*‐methylenebis(acrylamide) (BIS, Sigma, USA, cat# 146072) was set as 98:2, and the total concentration was 140 mm. Moreover, 0.1 mm fluorescein O‐acrylate (FL., Sigma, USA, cat# 568856) was added to impart visualization of the nanogels under confocal fluorescent microscopy with a Nikon Eclipse Ti inverted microscope (Nikon, Japan). The prescribed amount of NIPMAM and BIS, and 8 mm surfactant of sodium dodecyl sulfate (SDS, TCI, Japan, cat# I0352) were added to 100 mL deionized water (D. I. H_2_O) under stirring for 20 min to obtain a homogeneous solution. The solution was then transferred to a reaction flask after filtration and heated to 70 ˚C under a N_2_ atmosphere. The FL. was added at 70 ˚C and stirred for 20 min until the solution presented a light green color. When the temperature was kept stable at 70 ˚C for 20 min more, the polymerization was initiated by the injection of 1 mL of 800 mm ammonium persulfate (APS, TCI, Japan, cat# A2098) solution and lasted for 4 h under a N_2_ atmosphere. The product was filtered via a 0.22 µm syringe filter to remove any coagulum.

Subsequently, the nanogel cores were utilized as seeds for the growth of polymer shells through seeded precipitation polymerization. Briefly, NIPMAM, BIS, and *N*‐(3‐aminopropyl methacrylate hydrochloride) (APMA, Aladdin, China, cat# C2115084) were dissolved in 118.5 mL D. I. H_2_O at the molar ratio of 97.5:2:0.5 to prepare a monomer solution with the total concentration of 50 mm. A 30 mL nanogel core solution and 0.1721 g SDS were added to the reaction flask and preheated to 70 ˚C under a N_2_ atmosphere for 20 min. Then, the prepared monomer solution was transferred to the flask under vigorous stirring. After kept in a constant temperature at 70 ˚C for 20 min, the polymerization was initiated by the injection of 1.5 mL of 0.05 M APS solution and lasted for 4 h under a N_2_ atmosphere. The reaction was terminated via cooling down the system. The product was first filtered via a 0.22 µm syringe filter and then dialyzed for purification using a dialysis bag (MWCO: 12–14 kDa, Spectra/Por, Avantor, USA) for 3 days. The purified solution was lyophilized to obtain the core/shell nanogel powders, which were stored at 4 ˚C until use.

### Characterization of Core/Shell PNIPMAM Nanogel

The UV–vis spectrum of the prepared nanogel was measured using a UV–vis spectrometer (UV‐3600 Plus, Shimadzu, Japan). The lyophilized nanogel particles were dissolved in phosphate buffer saline (PBS, 1×, pH 7.4) to prepare a transparent suspension at the concentration of 3 mg mL^−1^. The UV–vis spectrum was recorded from 600 to 195 nm at a scanning rate of 0.5 nm s^−1^.

The size distribution of the thermo‐responsive PNIPMAM nanogel was determined via a dynamic light scattering analyzer (ZetaSizer Nano ZS90, Malvern Panalytical, Malvern, UK) from 25 to 41 ˚C. The lyophilized nanogel particles were dispersed in D. I. H_2_O and ultrasonicated for 10 min to prepare a transparent suspension at the concentration of 5 mg mL^−1^. The suspension was put into the chamber and allowed to equilibrate for 300 s before each measurement. The measurements were performed at a fixed scattering angle of 90˚. Particle zeta potential was measured using the same instrument at 37 ˚C. To confirm the nanogel size stability within the environments similar to in vivo, the size distributions of nanogel particles were recorded in D. I. H_2_O, PBS (pH 7.40), and 10% FBS (Thermo Fisher Scientific, USA, cat# 10270106) under 37 ˚C for 0, 1, and 3 days. All measurements were repeated 3 times. The average particle size was calculated based on the intensity distribution profile. Nanoparticle tracking analysis (NTA) measurements of the nanogel particles in D. I. H_2_O, PBS, and 10% FBS were performed with a NanoSight LM10 (NanoSight, Amesbury, UK) at 37 ˚C. The video frame of nanogel particles in different aqueous environments was extracted via the ImageJ software (version 1.52k).

The nanogel particle morphology was scanned using transmission electron microscopy (TEM, Tecnai G2 Spirit Bio, FEI, USA). First, the lyophilized nanogel particles were suspended in absolute ethanol and subsequently dropped onto the carbon‐coated copper grid. When the nanogel dried in the air, we performed TEM observation at the accelerated voltage of 120 kV.

### RGD Peptide Conjugation

The core/shell nanogel was modified with RGD peptide on the nanogel shell through the EDC coupling of FITC‐modified RGD peptide (Gly‐Arg‐Gly‐Asp‐Thr‐Pro, >98%, NJPeptide, Nanjing, China) to the primary amines. In brief, 27.6 mg nanogel particles were dissolved in 6 mL *N,N*‐dimethyl formamide (DMF, Merck, USA, cat# 200‐679‐5) to prepare a homogeneous suspension. 1 mg FITC‐modified RGD peptide, 0.264 mg 1‐(3‐dimethylaminopropyl)‐3‐ethylcarbodiimide hydrochloride (EDC · HCl, TCI, Japan, cat# D1601), and 0.16 mg *N*‐Hydroxysuccinimide (NHS, Sigma, USA, cat# 130672) were dissolved in 6 mL DMF and shaken for 30 min at room temperature to activate the carboxy acid group of RGD peptide. Then, this activated RGD solution was added to the nanogel suspension and reacted on a shaker at room temperature overnight. After the reaction, the product was filtered for purification via a 0.22 µm syringe filter and then dialyzed using a dialysis bag (MWCO: 12–14 kDa, Spectra/Por, Avantor, USA) for 3 days. The purified solution was lyophilized to obtain the RGD‐conjugated core/shell nanogel (^RGD−^Nanogel) powders, which were stored at 4 ˚C until use.

### Cellular Uptake of Nanogel and RGD−Nanogel Particles In Vitro

Cellular uptake of the nanoparticles was performed using 3T3 fibroblasts (ATCC, USA) and murine lung endothelial cells (provided by Professor Xiaohua Jiang's team, School of Biomedical Sciences, The Chinese University of Hong Kong, Hong Kong) according to the protocol.^[^ ^67^ ^]^ 1×10^5^ cells were seeded on the confocal dish (SPL Life Sciences, Korea). Nanogel or ^RGD−^Nanogel particles were added into media with a concentration of 250 µg mL^−1^. After incubation for 6 h, the cells were fixed with 3.7% formaldehyde (Sigma, USA, cat# 47608) for 10 min and washed cells with PBS for 3 times. Subsequently, the nuclei were stained with DAPI (Thermo Fisher Scientific, USA, cat# D1306) for 5 min. Cells were imaged with a Nikon Eclipse Ti inverted microscope (Nikon, Japan) at excited wavelengths of 408 nm (blue, nuclei) and 488 nm (green, nanogel, or ^RGD−^Nanogel). Images were digitalized by a *Z*‐stack mode with a step size of 0.33 µm.

### Live/Dead Assay

NIH3T3 cells were seeded onto a 6‐well plate at a density of 15 000 cells/cm^2^. Then, non‐functionalized nanogel particles were dispersed into the medium with a concentration of 250 µg/mL. The NIH3T3 cells were rinsed twice with PBS after incubation for 1 and 3 days, respectively. Then, the cells were stained with the LIVE/DEAD cell imaging kit (Thermo Fisher Scientific, USA, cat# R37601) according to the manufacturer's instructions. The cells were imaged with an inverted microscope (Nikon, Japan) at the wavelengths of 488 nm (green, indicating live cells) and 570 nm (red, indicating dead cells).

### IVIS Imaging for Tracing the IPFP‐Targeting Efficiency of RGD−Nanogel In Vivo

Mice at week 2 post‐sham or ‐DMM surgery were anesthetized by mask inhalation of isoflurane vaporized at concentrations of up to 4% in the induction phase and at 0.8–1.3% during prolonged experimental observation. The knee of the mice was gently shaved, and the skin was cleaned with betadine. Nanogel or ^RGD−^Nanogel solution (15 µL, 16 mg mL^−1^) was injected into the subcapsular IPFP without a surgical incision via 29G needle. As the nanogel was dressed by FITC‐modified RGD peptide to the primary amines, it was capable of imaging fluorescence (FITC signals) to render the retention of ^RGD−^Nanogel in vivo. An in vivo imaging system (IVIS200 imaging system, Xenogen Imaging Technologies, Alameda, CA, USA) was used accordingly to obtain images and quantify the signal intensity at 0‐, 1‐, 3‐, 6‐, 24‐, and 48‐hour post‐injections.

### Cryosection of IPFP

IPFP was collected and fixed in 4% PFA for 24 h at room temperature. Then, the tissue was dehydrated with 30% sucrose for 24 h and embedded in optimal cutting temperature (OCT, Tissue‐Tek O.C.T. Compound) compound. Sections (14 µm thick) were washed with distilled water and mounted with DAPI. Sections were scanned and digitalized with a microscopic imaging system.

### siRNA Encapsulation

The encapsulation of small interfering RNA (siRNA) by ^RGD−^Nanogel was achieved through a “breathing‐in” method according to the protocol.^[^ ^57^, ^66^, ^68^ ^]^ Briefly, the siRNA against *integrin β3* (*Cd61*, Thermo Fisher Scientific, USA, cat# AM16708) was suspended in nuclease‐free sterile water to prepare a siRNA solution at the concentration of 20 µm. Subsequently, the lyophilized ^RGD−^Nanogel particles were resuspended in siRNA solution with a concentration of 16 mg mL^−1^ and shaken at room temperature for 24 h.

The encapsulation efficiency of ^RGD−^Nanogel/siRNA solution was determined by measuring the siRNA concentration in the filtrate via UV–vis spectroscopy (UV‐3600 Plus, Shimadzu, Japan). Briefly, the lyophilized ^RGD−^Nanogel particles were resuspended in 0.5 µm siRNA solution as previously described. The suspension was loaded into a centrifugal filter tube (MWCO: 50 kDa, Amicron, Merck Millipore, USA) and centrifuged for 10 min at 4000 g. The filtrate was collected. The siRNA concentrations of the loading solution were determined and filtrated using the maximum absorbance value at 260 nm with UV–vis spectra, recording from 200 to 290 nm at a scanning rate of 0.5 nm s^−1^. The concentrations of siRNA in the loading solution and filtrate were calculated from a separately constructed standard curve of absorbance (at 260 nm) versus concentration (R^2^ > 0.99). The encapsulation efficiency (EE) was calculated by the following equation (Equation 1), where *c*_siRNA, loading_ denoted the concentration of siRNA in the loading solution, while *c*_siRNA, filtrate_ represented the concentration of siRNA in the filtrate.

(1) $$\mathrm{EE}\;=\;\frac{c_{\mathrm{siRNA},\;\mathrm{loading}}-c_{\mathrm{siRNA},\;\mathrm{filtrate}}}{c_{\mathrm{siRNA},\;\mathrm{loading}}}\times 100\%$$

### RGD−Nanogel siRNA Cd 61 Preparation before Injection

The lyophilized ^RGD−^Nanogel particles were diluted into the siRNA solution (20 µm) at a concentration of 16 mg mL^−1^ and shaken for 24 h, then stored at 4 °C in dark until use. ^RGD−^Nanogel siRNA *Cd61* solution was diluted for 7.5 folds with sterile PBS before injection.^[^ ^69^ ^]^ The control group was transfected with negative control siRNA (Thermo Fisher Scientific, USA, cat# AM4611). Transfection efficiency was assessed by measuring the expression of integrin *β*3 in IPFP after transfection in vivo.

### Intra‐IPFP Injection RGD−Nanogel siRNA Cd 61

For ^RGD−^nanogel siRNA *Cd 61* treatment in DMM group, anesthesia was maintained by mask inhalation of isoflurane vaporized at concentrations of up to 4% in the induction phase and at 0.8–1.3% during prolonged experimental observations. The knee joint hair of the mice was gently shaved, and the skin was treated with betadine. Mice were weekly received 15 µL of PBS, ^RGD−^Nanogel, ^RGD−^Nanogel‐(‐) siRNA *Cd 61*, or ^RGD−^Nanogel‐(+) siRNA *Cd 61* via a 29G needle into the subcapsular IPFP without a surgical incision from day 8 post‐surgery.^[^ ^69^ ^]^ All mice were returned to the cages when they were awake. Mice received seven intra‐IPFP injections in total and then were sacrificed by intraperitoneal injection of an overdose of sodium pentobarbital at week 8 post‐surgery. The knee joint was collected for histological and biological analysis.

### RNA Extraction of Articular Cartilage and IPFP from Mice

To improve the purity of tissue extraction, separation of IPFP or articular cartilage depot was done under a stereo zoom microscope using ophthalmic instruments.

Briefly, assembled IPFPs from six mice as one was incubated in Liberase (0.14 units mL^−1^, Roche, Switzerland, cat# 5401020001) for 30 min at 37 °C. After centrifugation for 5 min at 500 g, the floc at the bottom was regarded as the stromal vascular fraction (SVF), and the floating cells were regarded as the adipose tissue. The floating cells were digested for another 30 min. These repeat steps were aborted until no precipitation occurred. The pellets were collected for RNA extraction.

Cartilage depots were harvested from the medial tibial plateau and lowest point of the medial femoral condyle. Assembled cartilage tissues from four mice as one was digested with 0.2% collagen II for 30 min at 37 °C. Following centrifugation for 5 min at 500 g, the cell pellets were transferred into TRIzol reagent (Invitrogen, USA, cat# 15596026) for 30 min on ice.

### Quantitative Real‐Time Polymerase Chain Reaction (qRT‐PCR)

Harvested tissues were lysed in TRIzol reagent for total RNA extraction.^[^ ^70^ ^]^ The RNA sample was spectrophotometrically quantified using NanoDrop analysis (NanoDrop 2000/2000c Spectrophotometers, Thermo Fisher Scientific, USA, cat# ND‐2000). Total RNA was reverse‐transcribed to cDNA using a cDNA kit (Takara, USA, cat# 9160). cDNA was amplified in TB Green qPCR SuperMix‐UDG (Takara, USA, cat# RR420A) system containing specific primer sequences (Table S1, Supporting Information). The qRT‐PCR was run on a QuantStudio 12K Flex Real‐time PCR system (Life Technologies, Thermo Fisher Scientific, USA). Melting curve analysis was generated to determine the specificity of each qRT‐PCR reaction. The relative expression of mRNA level was calculated by the 2^−△△Ct^ method after normalizing to the house‐keeping gene (*Glyceraldehyde 3‐phosphate dehydrogenase, Gapdh*).

### Statistical Analysis

All treatments and assessments were not blinded, yet the results were confirmed and agreed by three independent investigators. All experiments have been repeated for at least three times. Quantitative data for each measurement was expressed as mean ± standard deviation (SD). *p* < 0.05 was statistically significant. Statistical analysis was performed using one‐way ANOVA with *Tukey's post hoc* test or two‐way ANOVA with *Tukey's post hoc* test for multiple group comparisons. For the longitudinal measures (for example Figure. 1a,b), two‐way ANOVA with repeated measures was conducted, followed by *Sidak's post hoc* test. One‐way ANOVA with *Dunnett's post hoc* test was used to compare the differences among treatment groups and the control group. A *two‐tailed Welch's t‐test* was used to test the difference between two groups. GraphPad Prism (version 8.2.1) was used for all statistical analyses.

## Conflict of Interest

The authors declare no conflict of interest.

## Author Contributions

B.D., Y.W.Z., J.X., T.N., and L.Q. conceptualized and designed. B.D., Y.W.Z., X.L., Z.L., S.X., S.Z., Z.Z., S.B., W.T., M.C., Y.L., X.Z., W.L., Y.T.Z., L.C., P.Y., K.H., T.N., J.X., and L.Q. performed experiments and data analysis. B.D., Y.W.Z., T.N., J.X., and L.Q. wrote the article with contributions from other authors.

## Supporting information

Supporting Information

## Data Availability

The data that support the findings of this study are available in the supplementary material of this article.
