# Supplementary material for: Blockage of Osteopontin‐Integrin β3 Signaling in Infrapatellar Fat Pad Attenuates Osteoarthritis in Mice
Source: Adv Sci (Weinh). 2023 May 23;10(22):2300897. doi: 10.1002/advs.202300897 (PMC10401113; doi:10.1002/advs.202300897)
Supplement: Supplementary file 1 — Supporting Information [file ADVS-10-2300897-s001.pdf]

ADVANCED SCIENCE
Open Access

Supporting Information

for *Adv. Sci.*, DOI 10.1002/advs.202300897

Blockage of Osteopontin-Integrin β3 Signaling in Infrapatellar Fat Pad Attenuates Osteoarthritis in Mice

Bingyang Dai, Yuwei Zhu, Xu Li, Zuru Liang, Shunxiang Xu, Shian Zhang, Zhe Zhang, Shanshan Bai, Wenxue Tong, Mingde Cao, Ye Li, Xiaobo Zhu, Wei Liu, Yuantao Zhang, Liang Chang, Patrick Shu-hang Yung, Kevin Ki-wai Ho, Jiankun Xu, To Ngai* and Ling Qin**

Supplementary Information

Blockage of osteopontin-integrin β3 signaling in infrapatellar fat pad attenuates osteoarthritis in mice

Dai *et al.*

Fig. S1. H&E staining of inflamed synovium.

Fig. S2. Representative images of immunofluorescence staining of F4/80 in IPFP.

Fig. S3. Illustration of the surgical access into mouse knee joint cavity.

Fig. S4. Illustration of the region of interest (ROI) for measuring the thickness of articular cartilage.

Fig. S5. Demonstration of the ROI for μCT analysis.

Table S1. Primer sequences used for qRT-PCR.

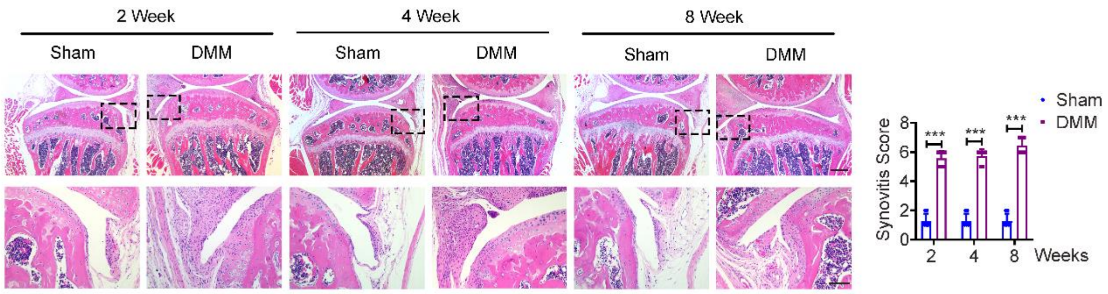

Fig. S1. H&E staining of inflamed synovium.

Representative H&E staining and synovitis scores of knee joint sections from sham and DMM mice at weeks 2, 4, and 8. Scale bar: 300 μm. The bottom row was magnified from the dotted frame in the top row. $n = 7$ mice per group. Images are representative of three independent experiments. All data are presented as mean ± SD. Two-way ANOVA with *Sidak's post hoc* test were used. ***$P < 0.001$.

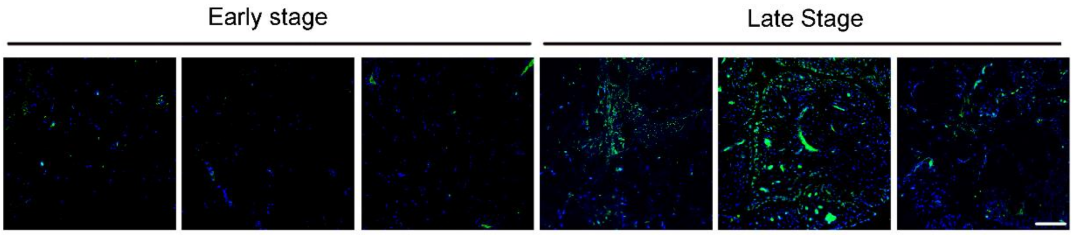

Fig. S2. Representative images of immunofluorescence staining of F4/80 in IPFP.

Representative images of immunofluorescence staining of F4/80 in IPFP from ACLR patients at early-stage (1-2 months of injury period) and late-stage (5-6 months of injury period). Scale bar: 100 μm. Images are representative of three independent experiments.

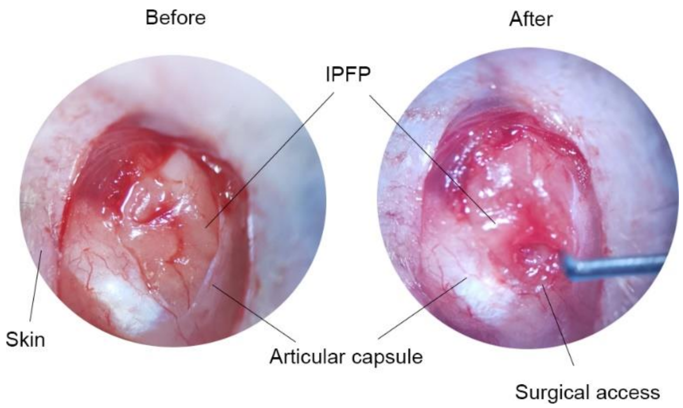

Fig. S3. Demonstration of the surgical access into mouse knee joint cavity.

The left picture showed the knee joint cavity before DMM surgery, and the right picture showed the surgical access into the articular cavity through infrapatellar fat pad (IPFP).

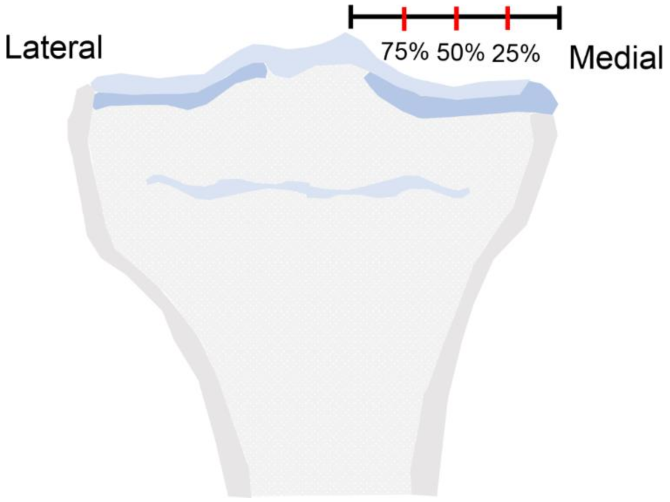

Fig. S4. Schematic illustration of the region of interest (ROI) for measuring the thickness of articular cartilage.

Three sections of each sample, echoing to 25%, 50%, and 75% of the tibial plateau from the medial collateral ligament side to medial intercondylar nodes, were stained with safranin O/fast green.

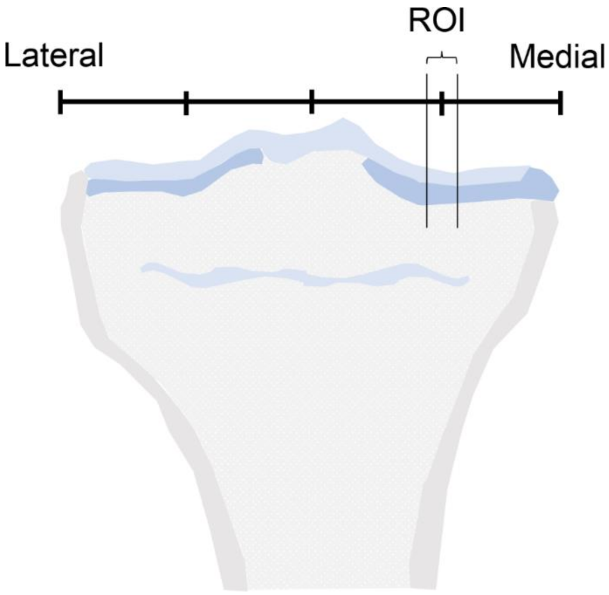

Fig. S5. Schematic illustration of the ROI for μCT analysis.

The ROI (width: 160 μm) for μCT analysis was the subchondral trabecular bone between subchondral cortical bone and growth plate at the quarter point of the tibial plateau from the medial collateral ligament side.

Table S1. Primer sequences used for qRT-PCR

Gene name	Oligonucleotide primers
Spp1-F	AGCAAGAAACTCTTCCAAGCAA
Spp1-R	GTGAGATTCGTCAGATTCATCCG
Il-1a-F	CGAAGACTACAGTTCTGCCATT
Il-1a-R	GACGTTTCAGAGGTTCTCAGAG
Il-1b-F	TTCAGGCAGGCAGTATCACTC
Il-1b-R	GAAGGTCCACGGGAAAGACAC
Il-4-F	GGTCTCAACCCCCAGCTAGT
Il-4-R	GCCGATGATCTCTCTCAAGTGAT
Il-5-F	CTCTGTTGACAAGCAATGAGACG
Il-5-R	TCTTCAGTATGTCTAGCCCCTG
Il-6-F	TAGTCCTTCCTACCCCAATTTC
Il-6-R	TTGGTCCTTAGCCACTCCTTC
Il-10-F	GCTCTTACTGACTGGCATGAG
Il-10-R	CGCAGCTCTAGGAGCATGTG
Il-13-F	CCTGGCTCTTGCTTGCCTT
Il-13-R	GGTCTTGTGTGATGTTGCTCA
Mcp1-F	CAGCCAGATGCAGTTAACGC
Mcp1-R	GCCTACTCATTGGGATCATCTTG
Nos2-F	GTTCTCAGCCCAACAATACAAGA
Nos2-R	GTGGACGGGTCGATGTCAC
Adipoq-F	TGTTCCTCTTAATCCTGCCCA
Adipoq-R	CCAACCTGCACAAGTTCCCTT

Tnfa-F	CCTGTAGCCCACGTCGTAG
Tnfa-R	GGGAGTAGACAAGGTACAACCC
Mmp2-F	CGATGTCGCCCCTAAAACAG
Mmp2-R	GCATGGTCTCGATGGTGTTC
Mmp3-F	GGCCTGGAACAGTCTTGGC
Mmp3-R	TGTCCATCGTTCATCATCGTCA
Mmp9-F	CTGGACAGCCAGACACTAAAG
Mmp9-R	CTCGCGGCAAGTCTTCAGAG
Mmp13-F	TGTTTGCAGAGCACTACTTGAA
Mmp13-R	CAGTCACCTCTAAGCCAAAGAAA
Mmp16-F	AGAAGGTTGGATTTCGTGCAT
Mmp16-R	TCCGCAGACTGTAGCACATAA
Vegfa-F	GCACATAGAGAGAATGAGCTTCC
Vegfa-R	CTCCGCTCTGAACAAGGCT
Leptin-F	GAGACCCCTGTGTCGGTTC
Leptin-R	CTGCGTGTGTGAAATGTCATTG
Actb-F	GGCTGTATTCCCCTCCATCG
Actb-R	CCAGTTGGTAACAATGCCATGT
Gapdh-F	AGGTCGGTGTGAACGGATTTG
Gapdh-R	TGTAGACCATGTAGTTGAGGTCA
Retnla-F	CCAATCCAGCTAACTATCCCTCC
Retnla-R	ACCCAGTAGCAGTCATCCCA
Cfd-F	CATGCTCGGCCCTACATGG
Cfd-R	CACAGAGTCGTCATCCGTCAC

Lamc1-F	TGCCGGAGTTTGTTAATGCC
Lamc1-R	CTGGTTGTTGTAGTCGGTCAG
Itgam-F	CCATGACCTTCCAAGAGAATGC
Itgam-R	ACCGGCTTGTGCTGTAGTC
Adgre1-F	TGACTCACCTTGTGGTCCTAA
Adgre1-R	CTTCCCAGAATCCAGTCTTTCC
Ctnnb1-F	ATGGAGCCGGACAGAAAAGC
Ctnnb1-R	CTTGCCACTCAGGGAAGGA
Mrc1-F	CTCTGTTCAGCTATTGGACGC
Mrc1-R	CGGAATTTCTGGGATTCAGCTTC
Itga5-F	CTTCTCCGTGGAGTTTTACCG
Itga5-R	GCTGTCAAATTGAATGGTGGTG
Fn1-F	TTCAAGTGTGATCCCCATGAAG
Fn1-R	CAGGTCTACGGCAGTTGTCA
Sparc-F	GTGGAAATGGGAGAATTTGAGGA
Sparc-R	CTCACACACCTTGCCATGTTT
Runx2-F	TTCAACGATCTGAGATTTGTGGG
Runx2-R	GGATGAGGAATGCGCCCTA
Timp1-F	GCAACTCGGACCTGGTCATAA
Timp1-R	CGGCCCGTGATGAGAAACT
Timp2-F	TCAGAGCCAAAGCAGTGAGC
Timp2-R	GCCGTGTAGATAAACTCGATGTC
Timp3-F	CTTCTGCAACTCCGACATCGT
Timp3-R	GGGGCATCTTACTGAAGCCTC

Timp4-F	TGTCTACACGCCATTTGACTC
Timp4-R	TGGACATCTCCTTACTTGGCA
Col1a1-F	GCTCCTCTTAGGGGCCACT
Col1a1-R	CCACGTCTCACCATTGGGG
Col1a2-F	GTAACTTCGTGCCTAGCAACA
Col1a2-R	CCTTTGTCAGAATACTGAGCAGC
Col3a1-F	CTGTAACATGGAAACTGGGGAAA
Col3a1-R	CCATAGCTGAACTGAAAACCACC
Col6a1-F	CTGCTGCTACAAGCCTGCT
Col6a1-R	CCCCATAAGGTTTCAGCCTCA
Col10a1-F	TTCTGCTGCTAATGTTCTTGACC
Col10a1-R	GGGATGAAGTATTGTGTCTTGGG
